# The LuxR Regulators PcoR and RfiA Co-regulate Antimicrobial Peptide and Alginate Production in *Pseudomonas corrugata*

**DOI:** 10.3389/fmicb.2018.00521

**Published:** 2018-03-23

**Authors:** Grazia Licciardello, Andrea Caruso, Patrizia Bella, Rodolpho Gheleri, Cinzia P. Strano, Alice Anzalone, Emmanouil A. Trantas, Panagiotis F. Sarris, Nalvo F. Almeida, Vittoria Catara

**Affiliations:** ^1^Parco Scientifico e Tecnologico della Sicilia, Catania, Italy; ^2^Dipartimento di Agricoltura, Alimentazione e Ambiente, Università degli Studi di Catania, Catania, Italy; ^3^Dipartimento di Scienze Agrarie, Alimentari e Forestali, Università degli Studi di Palermo, Palermo, Italy; ^4^School of Computing, Federal University of Mato Grosso do Sul, Campo Grande, Brazil; ^5^Dipartimento di Agraria, Università degli Studi “Mediterranea” di Reggio Calabria, Reggio Calabria, Italy; ^6^Department of Agriculture, School of Agriculture, Food and Nutrition, Technological Educational Institute of Crete, Heraklion, Greece; ^7^Department of Biosciences, College of Life and Environmental Sciences, University of Exeter, Exeter, United Kingdom; ^8^Institute of Molecular Biology and Biotechnology, Foundation for Research and Technology – Hellas, Heraklion, Greece

**Keywords:** cyclic lipopeptides, RNA-seq, non-ribosomal peptides, transcriptional analysis, exopolysaccarides

## Abstract

Cyclic lipopeptides (CLPs) are considered as some of the most important secondary metabolites in different plant-associated bacteria, thanks to their antimicrobial, cytotoxic, and surfactant properties. In this study, our aim was to investigate the role of the Quorum Sensing (QS) system, PcoI/PcoR, and the LuxR-type transcriptional regulator RfiA in CLP production in the phytopatogenic bacterium, *Pseudomonas corrugata* based on our previous work where we reported that the *pcoR* and *rfiA* mutants were devoid of the CLPs cormycin and corpeptin production. Due to the close genetic link between the QS system and the RfiA (*rfiA* is co-transcribed with *pcoI*), it was difficult to ascertain the specific regulatory role in the expression of target genes. A transcriptional approach was undertaken to identify the specific role of the PcoR and RfiA transcriptional regulators for the expression of genes involved in CLP production. The RNA-seq-based transcriptional analysis of the wild-type (WT) strain CFBP 5454 in comparison with GL2 (*pcoR* mutant) and GLRFIA (*rfiA* mutant) was performed in cultural conditions favoring CLP production. Differential gene expression revealed that 152 and 130 genes have significantly different levels of expression in the *pcoR* and *rfiA* mutants, respectively. Of these, the genes linked to the biosynthesis of CLPs and alginate were positively controlled by both PcoR and RfiA. Blast homology analysis showed that 19 genes in a large CLP biosynthetic cluster involved in the production of three antimicrobial peptides, which span approximately 3.5% of the genome, are strongly over-expressed in the WT strain. Thus, PcoR and RfiA function mainly as activators in the production of bioactive CLPs, in agreement with phenotype analysis of mutants. RNA-seq also revealed that almost all the genes in the structural/biosynthetic cluster of alginate exopolysaccharide (EPS) are under the control of the PcoR–RfiA regulon, as supported by the 10-fold reduction in total EPS yield isolated in both mutants in comparison to the parent strain. A total of 68 and 38 gene expressions was independently regulated by PcoR or RfiA proteins, respectively, but at low level. qPCR experiments suggest that growth medium and plant environment influence the expression of CLP and alginate genes.

## Introduction

*Pseudomonas corrugata* Roberts and Scarlett 1981 is a ubiquitous bacterium in agro-ecosystems. It has been isolated from bulk soils, plant rhizosphere, and either as endophyte or parasite from diverse cultivated plants (Catara, 2007). It was first described in the United Kingdom (Scarlett et al., 1978) as the causal agent of tomato pith necrosis (TPN) and later was reported in association with TPN worldwide (Catara, 2007). It has a very low host range, and along with tomato, it has been sporadically described as a plant pathogen on pepper and chrysanthemum (Catara, 2007). *P. corrugata* strains have a notable antimicrobial activity against bacteria, chromista, and fungi, and some strains have been successfully tested as biological control agents in different pathosystems (Catara, 2007; Strano et al., 2017). *P. corrugata* produces several bioactive compounds such as the lipopeptide siderophore corrugatin (Risse et al., 1998), the cyclic lipopeptides (CLPs) cormycin A and corpeptin A and B (Emanuele et al., 1998; Scaloni et al., 2004), and hydrogen cyanide (Strano et al., 2017). Cormycin and corpeptins merit great interest as they have both phytotoxic and antimicrobial properties (Emanuele et al., 1998; Scaloni et al., 2004). CLPs consist of a short oligopeptide that is cyclized to form a lacton ring with a linked fatty acid tail and they may have diverse roles in plant-associated *Pseudomonas* species, such as motility, biofilm formation, antimicrobial activity, and they also play a key role in virulence of phytopathogenic bacteria (Bender et al., 1999; Raaijmakers et al., 2006).

Cyclic lipopeptides are synthesized with a thiotemplate process by large multifunctional non-ribosomal peptide synthetases (NRPSs) that have a modular structure (Gross and Loper, 2009). Each module serves as a building block for the gradual incorporation of one amino acid in the peptide chain (Finking and Marahiel, 2004; Raaijmakers et al., 2006). CLP biosynthetic loci, organized in clusters which include transporter systems and regulatory genes, have been described in several *Pseudomonas* spp. (de Bruijn and Raaijmakers, 2009a; Gross and Loper, 2009). Proteins of the LuxR superfamily play an important role in the regulation of CLP production. This superfamily consists of transcriptional regulators containing a DNA-binding helix-turn-helix (HTH) motif in the C-terminal region and the proteins are grouped into different subfamilies based on their domain architecture and activation mechanism (Subramoni and Venturi, 2009; Chen and Xie, 2011; Vaughn and Gross, 2016). Three different LuxR-type regulators are involved in CLP biosynthesis in many *Pseudomonas* species (Lu et al., 2002; Dubern et al., 2005; Wang et al., 2006a; Berti et al., 2007; de Bruijn and Raaijmakers, 2009b). The first group consists of regulators belonging to a two-component sensory transduction system, activated upon the phosphorylation of a transmembrane kinase as in the GacA/GacS regulatory system, with a key role in syringomycin–syringopeptin, putisolvins, massetolide A, and viscosin production (Scholz-Schroeder et al., 2001; Dubern et al., 2005; de Bruijn et al., 2007, 2008). Mutations disrupting either of the two genes result in impaired CLP production. The second group consists of regulators that contain an autoinducer-binding domain in the N-terminal region, usually activated via binding to an *N*-acyl homoserine lactone (AHL) in different *Pseudomonas* spp. (von Bodman et al., 2003; Venturi, 2006; Subramoni and Venturi, 2009). The conjugate serves as a signaling molecule involved in Quorum Sensing (QS). AHL-QS plays a role in CLP production in terms of viscosin and putisolvin biosynthesis in the plant pathogenic *P. fluorescens* strain 5064 and the saprophytic *P. putida* strain PCL1445, respectively (Cui et al., 2005; Dubern et al., 2006). The third group of LuxR-type transcriptional regulators harbors the typical C-terminal HTH DNA-binding domain but lacks an N-terminal regulatory domain. They have been found positioned up and downstream of the CLP biosynthesis clusters of different *Pseudomonas*, playing a crucial role in the production of several CLPs, including syringomycin, syringopeptin, syringafactins, putisolvins, viscosin, massetolide, sessilin, and orfamide (Lu et al., 2002; Dubern et al., 2005; Wang et al., 2006a; Berti et al., 2007; de Bruijn and Raaijmakers, 2009b; Vaughn and Gross, 2016; Olorunleke et al., 2017).

In our previous studies, we demonstrated that two LuxR-type regulators, PcoR and RfiA in *P. corrugata*, have a role in virulence on tomato, and elicitation of hypersensitive-like response on *Nicotiana* spp. Neither cormycin nor corpeptins were detected in the culture filtrates of the *pcoR* and *rfiA* mutants (Licciardello et al., 2012). However, only in the *rfiA* mutant was the ability to inhibit fungal growth in dual plate assays greatly reduced (Strano et al., 2017). PcoR is part of a QS system mediated by a set of AHLs, namely *N*-hexanoyl-L-homoserine lactone (C6-HSL), 3-oxo-C6-HSL, and C8-HSL, and it is synthesized thanks to the AHL synthase PcoI (Licciardello et al., 2007). Unlike PcoR, RfiA lacks a N-terminal regulatory domain but it is directly controlled by QS via positive-feedback regulatory loops, since *rfiA* is located downstream of *pcoI* and they are co-transcribed (Licciardello et al., 2009). The 20 kb cosmid insert in which QS genes were identified was also shown to contain an operon designated as *pcoABC* downstream of *rfiA*. This operon encodes a tripartite resistance nodulation-cell-division (RND) transporter system. Genes encoding for an ABC-transport system and part of an NRPS are involved in the production of corpeptins, designated as *crpCDE* (Licciardello et al., 2009; Strano et al., 2015). Hierarchical regulation where the PcoR–AHL complex regulates the *pcoI/rfiA* operon and, in turn, RfiA activates *pcoABC* transcription has been demonstrated (Licciardello et al., 2009). Since RfiA does not require AHL to be active, its complementation *in trans* has also been shown to be sufficient to restore pathogenicity of the *pcoR* mutant in the absence of AHL (Licciardello et al., 2009). Interestingly the *pcoI* mutant, which is actually a *pcoI*-/*rfiA*-double mutant, has been shown to be as virulent as the wild-type (WT) strain. Thus, it has been suggested a model where either QS regulates the synthesis of RfiA or PcoR regulates virulence independently of the AHL (Licciardello et al., 2009).

Genome analysis has revealed that *P. corrugata* could putatively produce at least four NR peptides, a polyketide, and a bacteriocin (Licciardello et al., 2014; Trantas et al., 2015). The availability of the genome sequences of a number of *P. corrugata* strains including our model strain CFBP 5454, isolated from tomato affected by TPN, led us to further investigate both the role of PcoR and RfiA by an RNA-seq approach. Under the experimental conditions proven to induce cormycin and corpeptin production *in vitro* (Scaloni et al., 2004; Licciardello et al., 2009; Strano et al., 2015) PcoR and RfiA positively regulate the same set of genes involved in the secondary metabolite production of (i) three antimicrobial peptides in a DNA region that spans approximately 3.5% of the *P. corrugata* genome and (ii) all of the biosynthetic/structural alginate genes. In line with these findings, supported by phenotypic analysis, in this work we further support the previously proposed model, for *pcoABC* regulation. In this model, QS at a high cellular concentration regulates these important traits for *P. corrugata* fitness and biology via RfiA. Expression analysis studies on the WT strain also suggest that in comparison to alginate genes, CLP genes present higher expression levels in minimal media, while alginate genes presented higher expression in rich media and *in planta*.

## Materials and Methods

### Bacterial and Fungal Strains and Routine Growing Conditions

*Pseudomonas corrugata* strain CFBP 5454 and the derivative mutants used in this study are listed in **Table 1**. They were routinely cultured at 28°C on either Nutrient Agar (NA, Oxoid, Milan, Italy) supplemented by 1% D-glucose (NDA), or Luria-Bertani (LB) agar (Oxoid, Milan, Italy) (**Table 1**). The *pcoR*-mutant strain, designated GL2 is a Tn5 mutant (*pcoR76::Tn5*) (Licciardello et al., 2007); the *rfiA* mutant (GLRFIA strain) was obtained by insertional mutagenesis using the conjugative suicide vector pKNOCK-Km (*rfiA::pKnock*) (Licciardello et al., 2009). The complemented mutant strains used in phenotypic tests are listed in **Table 1**.

**Table 1:** Bacterial strains and plasmids used in this study.

Strain, plasmid	Relevant characteristic^a^	Reference^b^
*P. corrugata* CFBP5454	WT, source of *pcoR* and *rfiA*	CFBP
*P. corrugata* GL2	*pcoR*76::Tn5 of CFBP 5454, Km^r^	Licciardello et al., 2007
*P. corrugata* GLRFIA	*rfiA*:: pKnock, Km^r^	Licciardello et al., 2009
*P. corrugata* GL2C	*P. corrugata* GL2 mutant complemented with cosmid pLC3.34, Tc^r^	Licciardello et al., 2007
*P. corrugata* GLRFIAC	*P. corrugata* GLRFIA mutant complemented with plasmid pBBR–RfiA, Gm^r^	Licciardello et al., 2009
*P. corrugata* GL2 + RfiA	*P. corrugata* GL2 mutant complemented with pBBR–RfiA, Gm^r^	Licciardello et al., 2009
pBBR–RfiA	pBBR1MCS-5 containing the full-length *P. corrugata* CFBP 5454 *rfiA* gene	Licciardello et al., 2009
pLC3.34	pLAFR3 containing *P. corrugata* CFBP 5454 DNA, Tc^r^	DISTEF
pLC3.34::Tn5-4	pLC3.34 with Tn5 insertion in pcoR, position 76, Tc^r^, Km^r^	Licciardello et al., 2007

*^*a*^Km^*r*^, Tc^*r*^, and Gm^*r*^ indicate resistance to kanamycin, tetracycline, and gentamicin, respectively. ^*b*^CFBP, Collection Francaise de Bacteries Phytopathogenes, Angers, France; DISTEF, Dipartimento di Scienze e Tecnologie Fitosanitarie, Catania, Italy; DISTEF, Dipartimento di Scienze e Tecnologie Fitosanitarie (now Di3A), University of Catania, Italy.*

Antibiotics were added as required in the following final concentrations: tetracycline, 40 μg ml^-1^; gentamicin, 40 μg ml^-1^, and kanamycin, 100 μg ml^-1^.

For transcript profiling by RNA-seq, mid-logarithmic phase cells grown on nutrient broth (NB, Oxoid, Milan, Italy) were used to inoculate Improved Minimal Medium (IMM) (Surico et al., 1988) at an OD_600_ = 0.05, and incubated in static conditions at 28°C (Scaloni et al., 2004; Licciardello et al., 2012; Strano et al., 2015). In each experiment three separate batch cultivations were performed for each bacterial strain. The Gram-positive bacterium *Bacillus megaterium* ITM100 and the yeast *Rhodotorula pilimanae* ATCC 26423 were used as bioindicators of CLP production according to Lavermicocca et al. (1997).

### RNA Isolation

RNA from WT *P. corrugata* CFBP 5454 as well as GL2 (*pcoR76::Tn5*) and GLRFIA (*rfiA::pKnock*) mutants were extracted from cells grown at the early stationary phase (*t* = 40 h, OD_600_ = 8.9) in IMM at 28°C. Samples from three replicates of each strain grown on separate days and different batches of medium were collected. The cultures were fixed using RNA^TM^ Protect Bacterial Reagent (Qiagen Inc.) in a ratio of 2 ml of reagent per 1 ml of bacterial culture. Centrifugation was used to pellet the cells (5000 rpm, 4°C, 20 min), and RNA was extracted in RNase-/DNase-free water using the RNeasy Mini Kit (Qiagen Inc.). Total RNA was quantified using micro-spectrophotometry (Nanodrop^TM^ 2000C, Thermo Scientific^TM^, Waltham, MA, United States). The RNA quality was estimated using an Agilent 2100 Bioanalyzer and RNA samples with an RNA Integrity Number (RIN) above 8.0 were selected.

### Library Construction and RNA Sequencing

Libraries were prepared for sequencing according to the manufacturer’s instructions (Illumina). Single-end 51 nucleotide sequence reads were obtained using the Illumina HiSeq2000 system at Parco Tecnologico Padano (Lodi, Italy), processed with Casava version 1.8. Raw sequencing reads were quality controlled using FastQC v.0.10.1 and processed with Trimmomatic v.0.32 to remove sequencing adapters and low-quality bases. High-quality filtered reads were aligned against the *P. corrugata* genome (ATKI01000000). Bowtie v2.2.2 software was used to perform the alignments and generate the corresponding BAM files.

Aligned reads were processed using HTSeq v0.6.1 to extract read counts over the annotated genes for the genome provided. For all samples, the number of raw reads mapping to each gene was normalized based on the total number of input reads (non-rRNA and non-tRNA reads) for that sample. This normalization procedure enabled gene-expression patterns to be compared across strains, within and between experiments. Reads that partially overlapped a gene contributed to its total raw read value. Only genes that had an average of >10 reads in the three replicates for the WT in comparison with the mutants were considered for further analyses.

The read counts for each sample were imported into R and processed using the Bioconductor package EdgeR. Counts values were normalized using the Trimmed Mean of M-values (TMM) method and statistical comparisons of expression levels across different groups were performed using the EdgeR exact test method. For the further analyses, genes with a false-discovery rate of ≤0.05 were selected. We relied on the top 243 differentially expressed genes without any fold change cut-off.

The RNA-Seq data were submitted to the Sequence Read Archive (SRA) under accession number SRP128274.

### RNA Isolation From Inoculated Tomato Plants

RNA was extracted from tomato cv. Bacio plants previously inoculated with *P. corrugata* CFBP 5454. Tomato plants were grown in nursery flats. After germination and during the trials, plants were maintained in a growth chamber with a 16 h/8 h photoperiod and a temperature of 26°C. Tomato plants were pin-pricked on the stem at the axil of the first true leaf with bacterial cells from 48-h culture on NDA (Licciardello et al., 2007). Four days after inoculation, 5 cm of stem portions including the inoculation site was sampled and stored at -80°C. Pools of four stems for each bacteria-inoculated plant were ground in liquid nitrogen and 100 mg of powder processed for total RNA extraction with the RNeasy Plant minikit (Qiagen Inc.), according to the manufacturer’s instructions.

### Primer Design and Quantitative Real-Time PCR Validation

Quantitative Real-Time PCR (qPCR) was performed on 13 genes of the CLP cluster (*crpC, grsb_1, grsB_2, dhbF_3, dhbF_4, syrD2, bepE_1, mefA, arpC, crpD, pcoA, pcoB, oprM_3*). Three genes belonging to the biosynthetic cluster of alginate (*algD, algG, algI*) were selected for validation too. Nucleotide FASTA sequences were retrieved from the *P. corrugata* CFBP 5454 genome and used to design the primer sets useful for qPCR. Primers were designed with Beacon software (Premier Biosoft International Ltd., Palo Alto, CA, United States) and validated by BLAST (Altschul et al., 1990) in order to minimize the mispriming sites in other genomic loci (Supplementary File 1).

After treatment of the RNA samples with DNAse I (Invitrogen, Life Technologies, Italy), 1 μg of total RNA (from three different independent extractions) was used for cDNA synthesis with Superscript III (Invitrogen, Life Technologies, Italy) according to the manufacturer’s protocol. Samples in which reverse transcriptase had not been added were used as negative controls.

Reactions were conducted with the BioRad iQ Cycler and the SYBR^®^ Select Master Mix for CFX (Applied Biosystem, Life Technologies, Italy) according to the manufacturer’s protocols. To correct small differences in template concentration, the 16S rRNA gene was used for normalization (Conte et al., 2006). Analysis of the dissociation curve ensured that a single product was amplified. cDNA synthesis reaction was performed at 95°C for 15 s, and at 58–64°C for 1 min (for annealing temperatures see Supplementary File 1).

Data were analyzed using the comparative Ct method, wherein the Ct values of the samples of interest were compared to the Ct values of a control. All the Ct values were normalized versus the 16S rRNA gene. The relative expression (RE) values were calculated by the formula RE = 2-[ΔCt(Wt) - ΔCt(mutant)] (Livak and Schmittgen, 2001).

### Bioinformatics Tools for Genomic and Transcriptomic Data

Genome comparative analysis and gene cluster visualization were performed using the Integrated Microbial Genomes & Microbiomes (IMG/M) system^1^. The antiSMASH software pipeline (Blin et al., 2013) was used for the automated identification of secondary metabolite biosynthesis clusters. The number of genes differentially regulated was shown in a Venn diagram. Graphical representation of the relationship between intensity (LogCPM) and difference (Log2FC) of transcripts between *P. corrugata* CFBP 5454 (Wt) versus GL2 and GLRFIA derivatives mutants was done graphically represented using the DEPICTViz, Differential Expression, and Protein InteraCTions Visualization tool (Lima et al., 2016). Functional annotation, which categorizes genes into functional classes, was performed by Gene Ontology (GO) identification developed at the GO Consortium (Ashburner et al., 2000).

### *In Vitro* Bioassay for CLP Production

Antimicrobial activity of 10× concentrated culture filtrates from bacterial strains grown in IMM and NB were assessed after 4 days of incubation in static conditions, using the well diffusion assay according to Licciardello et al. (2009). Two CLP-sensitive bioindicator strains *R. pilimanae* ATTC26432 and *B. megaterium* ITM100 previously grown in layers on top of agar potato dextrose agar plates (PDA, Oxoid, Milan, Italy) were used (Licciardello et al., 2009). The in zone for each antimicrobial compound tested was measured. All tests were carried out at least twice in triplicate.

### Exopolysaccharide Isolation and Quantification

Total exopolysaccharides (EPSs) were isolated from *P. corrugata* CFBP 5454 and derivative mutants grown in IMM at 28°C for 4 days. EPSs were also evaluated from WT strain grown on NB. After centrifugation at 16,300 × *g* for 20 min to remove cells, total EPSs were isolated according to Fett et al. (1996) with slight modifications (Licciardello et al., 2017). Three separate partially purified samples were prepared for each bacterial strain.

### Statistical Analysis

Data were analyzed by two-way ANOVA using IBM^®^ SPSS^®^ v20. Mean values were compared using the Student–Newman–Keuls test. Statistical significance was established at *P* ≤ 0.05 and *P* ≤ 0.001.

## Results

### Differential Expression Analysis of the Transcriptome of *Pseudomonas corrugata* CFBP 5454 Versus *pcoR* and *rfiA* Mutants

To investigate the regulatory functions of *P. corrugata* LuxR transcriptional regulators PcoR and RfiA, expression profiles from RNA-seq data were analyzed. The transcriptome of *P. corrugata* strain CFBP 5454 was compared to those of the mutant strains GL2 (*pcoR* mutant) and GLRFIA (*rfiA* mutant) (Licciardello et al., 2007, 2009) grown to the early stationary phase in IMM which facilitates CLP production (Scaloni et al., 2004; Licciardello et al., 2009, 2012). Libraries derived from single-stranded cDNAs were sequenced and mapped against *P. corrugata* CFBP 5454 reference genome (ATKI01000000). Genes with increased or decreased expression in the WT strain compared to the mutant strains were considered to be positively or negatively regulated by PcoR or RfiA.

**FIGURE 1:**
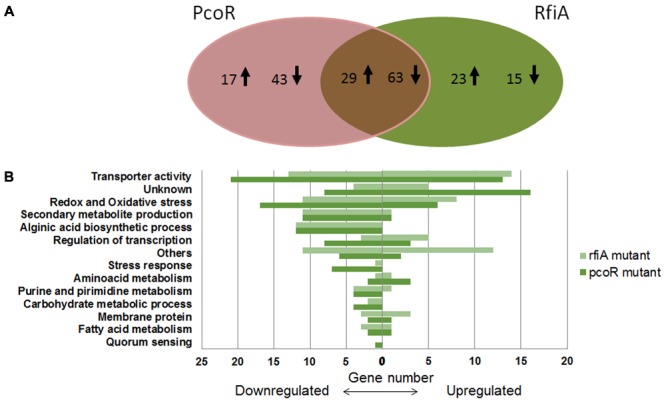
Differential expressed genes in *Pseudomonas corrugata* CFBP 5454 WT strain in comparison to *pcoR*- and *rfiA*-derivative mutants by RNA-seq analysis. **(A)** Venn diagram with up-/down-regulated genes after mutation in *pcoR* and/or *rfiA* genes. A total of 180 genes were found to be differentially expressed, 92 of which overlap in the transcriptional profiles of both mutants (in the center of the diagram). Up and down arrows represent genes up- and down-regulated, respectively. **(B)** Number and functional classification of genes up- and down-regulated in *P. corrugata* strain CFBP 5454 in comparison with *pcoR*- and *rfiA-*derivative mutants. The plot indicates the type of physiological role(s) and the total number of genes with decreased and increased expression within each category.

With a false-discovery rate (FDR) correction of 5%, 152 genes (46 increased and 106 decreased) differed significantly in the *pcoR* mutant, and 130 genes (52 increased and 78 decreased) differed significantly in the *rfiA* mutant compared to the parent strain CFBP 5454 (**Figure 1A**). Overall, the expression of 92 genes, which represent 3% of the annotated genes in the CFBP 5454 draft genome, differed significantly in both *pcoR* and *rfiA* mutants (Supplementary Files 2, 3). The remaining 60 (out of 152) and 38 (out of 130) genes were independently regulated either by PcoR or RfiA, respectively (**Figure 1A**) (Supplementary Files 4, 5). The Supplementary Files contain a thorough analysis of the transcripts and their predicted functions found to be associated with the role of PcoR and RfiA (Supplementary Files 2–5). In order to assemble a catalog of functions strongly linked to these transcriptional regulators, differentially expressed genes for both mutants were grouped based on their GO utilizing GO Consortium^2^. Genes were grouped into 14 functional categories on the basis of PseudoCAP and were plotted with respect to down-regulation and up-regulation (**Figure 1B**).

The largest group consisted of enzymes associated with transport systems, 34 of which were differentially expressed in the WT strain compared with expression in *pcoR* mutant and 27 with *rfiA* mutant. The second largest group were the genes involved in redox and oxidative stress, most of which were down-regulated in both *pcoR* (17 genes) and *rfiA* (11 genes) mutants. Genes predicted to be related with alginic acid biosynthesis (12 genes) and secondary metabolite production (11 genes) were well represented among the over-expressed genes in the WT strains in comparison to mutants, thus revealing the predominantly positive control of both PcoR and RfiA in the biosynthesis of these molecules. Transcriptional regulator genes account for a significant number of transcripts affected by *pcoR* and *rfiA* mutations, including up- (8 genes) and down- (11 genes) regulated genes that show a wide-ranging control through a cascade of other regulators. Other gene categories affected are involved in carbohydrate metabolic processes, fatty acids, amino acids, and purine and pyrimidine metabolisms.

### CLP Biosynthesis Clusters Are Part of the PcoR–RfiA Regulon

**FIGURE 2:**
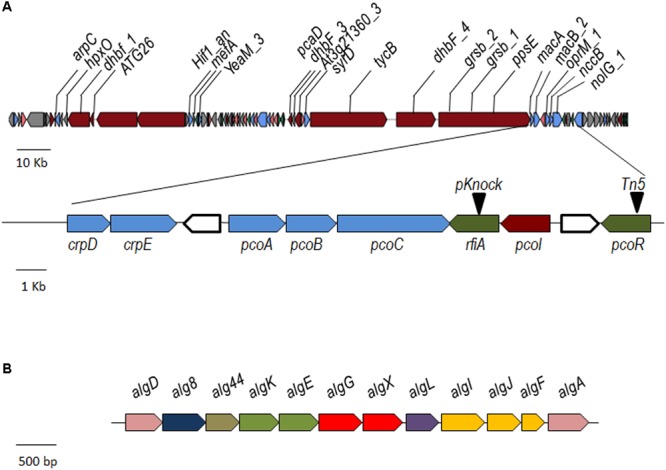
The *P. corrugata* gene clusters involved in the biosynthesis of cyclolipopeptides **(A)** and alginate **(B)**. **(A)** Genes over-expressed in *P. corrugata* CFBP 5454 in comparison with both *pcoR*- and *rfiA*-derivative mutants are labeled in the upper part of the graph. In the enlarged frame, genes for PcoI/PcoR QS system, RfiA, PcoABC, and CrpDE transporter system are labeled. Triangles indicate the positions of insertional mutagenesis. *pcoI* was over-expressed in the parent strain only in comparison to the *pcoR*-derivative mutant. Core biosynthetic genes (dark red), transport-related genes (light blue), regulatory genes (dark green), additional biosynthetic gene (pink), and other genes (gray) are represented. **(B)** Genes of the alginate structural/biosynthetic cluster differentially expressed in both *pcoR* and *rfiA* mutants; sugar production (pink), subunit polymerization (dark blue), c-di-GMP (gray), outer membrane secretion protein (light green), epimerase/modification (red), lyase (violet), and *O*-acetylation (yellow).

RNA-seq analysis showed that among the transcripts differentially expressed in both *pcoR* and *rfiA* mutants, there are 21 genes putatively involved in CLP production, as ascertained by homology BLAST analysis. The genome of *P. corrugata* CFBP 5454 was assembled into 156 contigs and NRP genes were located in at least 10 different contigs (Licciardello et al., 2014; Trantas et al., 2015). Therefore, for the *in-silico* reconstruction of *P. corrugata* secondary metabolite clusters, we used the annotated sequence of strain LMG 2172^T^ (also known as BS3649, Genbank accession NZ_LT629798.1) which shared an average nucleotide identity (ANI) of 99.53% with strain CFBP 5454. Using combined AntiSMASH and BLAST analyses, we found that the differentially expressed genes were located in a large HSL-NRPS cluster accounting for approximately 3.4% of the LMG 2172^T^ genome (**Figure 2A, Table 2** and Supplementary File 6).

**Table 2:** Locus tag of significantly differentially expressed genes in *P. corrugata* strain CFBP 5454 derivative mutants *pcoR* and *rfiA* and correspondent loci in strain LMG 2172^T^.

CFBP5454 locus tag	LMG2172^T^ locus tag^∗^	Putative gene	Description	Function	LogFC *pco*R mutant	LogFC *rfiA* mutant
K659_RS0103735	BLU14_RS07135	arpC	Antibiotic efflux pump outer membrane protein ArpC	Membrane protein	3.07	2.92
K659_RS0103725	BLU14_RS07145	hpxO	FAD-dependent urate hydroxylase	Purine and pirimidine	4.63	4.69
K659_RS0103720	BLU14_RS07150	dhbF_1	Dimodular nonribosomal peptide synthase	Secondary metabolite production	4.85	4.84
K659_RS0103715	BLU14_RS07155	ATG26	Sterol 3-beta-glucosyltransferase	Transporter activity	5.72	5.72
K659_RS0121340	BLU14_RS07170	Hif1 an	Hypoxia-inducible factor 1-alpha inhibitor	Redox and oxidative stress	3.95	4.01
K659_RS0121335	BLU14_RS07175	mefA	Macrolide efflux protein A	Transporter activity	3.49	3.68
K659_RS0121325	BLU14_RS07185	yeaM_3	Putative HTH-type transcriptional regulator YeaM	Regulation of transcription	3.05	2.93
K659_RS0123940	BLU14_RS07330	pcaD_2	3-Oxoadipate enol-lactonase 1	Secondary metabolite production	4.89	4.94
K659_RS0123930	BLU14_RS07340	dhbF_3	Dimodular nonribosomal peptide synthase	Secondary metabolite production	5.19	7.18
K659_RS0123920	BLU14_RS07345	At3g21360_3	Clavaminate synthase-like protein	Redox and oxidative stress	6.15	6.55
K659_RS0123925	BLU14_RS07350	syrD_2	ATP-binding protein SyrD	Secondary metabolite production	5.28	5.51
K659_RS01000000128675	BLU14_RS07355	tycB	Tyrocidine synthase 2	Secondary metabolite production	5.30	5.42
K659_RS01000000128480	BLU14_RS07360	dhbF_4	Dimodular nonribosomal peptide synthase	Secondary metabolite production	5.19	5.02
K659_RS01000000128500	BLU14_RS07365	grsB_2	Gramicidin S synthase 2	Secondary metabolite production	6.08	6.25
K659_RS0121920	BLU14_RS07365	grsB_1	Gramicidin S synthase 3	Secondary metabolite production	5.76	5.95
K659_RS0115225	BLU14_RS07365	ppsE_1	Plipastatin synthase subunit E	Secondary metabolite production	5.78	5.79
K659_RS0115230	BLU14_RS07370	macA	Macrolide export protein MacA	Transporter activity	5.85	5.69
K659_RS0115235	BLU14_RS07375	macB2	Macrolide export ATP-binding/permease protein MacB 2	Transporter activity	4.71	4.83
K659_RS0115245	BLU14_RS07385	oprM_1	Outer membrane protein OprM	Transporter activity	4.39	4.29
K659_RS0115250	BLU14_RS07390	nccB	Nickel–cobalt–cadmium resistance protein NccB	Stress response	3.52	3.83
K659_RS0115255	BLU14_RS07395	nolG_1	Nodulation protein NolG	Transporter activity	2.50	2.59
K659_RS0107190	BLU14_RS00885	algD	GDP-mannose 6-dehydrogenase	Alginic acid biosynthetic process	4.09	4.09
K659_RS0107185	BLU14_RS00880	alg8	Glycosyltransferase	Alginic acid biosynthetic process	2.70	2.75
K659_RS0107180	BLU14_RS00875	alg44	Alginate biosynthesis protein	Alginic acid biosynthetic process	3.09	3.30
K659_RS0107175	BLU14_RS00870	algK	Alginate biosynthesis protein	Alginic acid biosynthetic process	2.64	2.56
K659_RS0107170	BLU14_RS00865	algE	Alginate production protein	Alginic acid biosynthetic process	1.95	2.22
K659_RS0107165	BLU14_RS00860	algG	Poly(beta-D-mannuronate) C5 epimerase	Alginic acid biosynthetic process	1.93	2.00
K659_RS0107160	BLU14_RS00855	algX	Alginate biosynthesis protein AlgX	Alginic acid biosynthetic process	2.33	2.42
K659_RS0107155	BLU14_RS00850	algL	Alginate lyase	Alginic acid biosynthetic process	2.29	2.45
K659_RS0107150	BLU14_RS00840	algI_1	Putative alginate *O*-acetylase	Alginic acid biosynthetic process	2.27	2.15
K659_RS0107145	BLU14_RS00835	algJ_1	Putative alginate *O*-acetylase	Alginic acid biosynthetic process	2.28	2.43
K659_RS0107140	BLU14_RS00830	algF	Alginate biosynthesis protein	Alginic acid biosynthetic process	3.38	3.28
K659_RS0107135	BLU14_RS00825	algA	Alginate biosynthesis protein	Alginic acid biosynthetic process	3.05	2.98
K659_RS0104930	Not found	oprM_3	Outer membrane protein OprM	Transporter activity	-2.32	-2.13
K659_RS0104925	Not found	bepE_1	Efflux pump membrane transporter BepE	Transporter activity	-2.06	-2.03
K659_RS0120785	BLU14_RS16215	gph_2	Phosphoglycolate phosphatase	Carbohydrate metabolic process	3.15	3.34
K659_RS0120780	BLU14_RS16225	DIT1_2	Spore wall maturation protein DIT1	Unknown	3.21	3.52
K659_RS0120790	BLU14_RS16220	DIT1_1	Spore wall maturation protein DIT1	Unknown	3.30	3.57
K659_RS0104870	Not found	azoB_4	NAD(P)H azoreductase	Redox and oxidative stress	4.05	4.23
K659_RS0115240	BLU14_RS07380	rhbA_1	Diaminobutyrate-2-oxoglutarate aminotransferase	Secondary metabolite-1 production	4.42	4.81
K659_RS0111135	BLU14_RS25505	yddQ_1	Putative isochorismatase family protein YddQ	Others	4.63	5.09

This large cluster includes six NRPS genes most of which were putatively attributed to two closed CLP biosynthetic clusters for the synthesis of corpeptins, 22 amino acid lipopeptides, and the nonapeptide cormycin A. A similar topology was observed for nunapeptins and nunamycin in *P. fluorescens* In5 (Michelsen et al., 2015; Hennessy et al., 2017) and for thanapeptins, and thanamycin of *Pseudomonas* sp. SH-C52 (Mendes et al., 2011; Van Der Voort et al., 2015). In addition, in close proximity to the cormycin cluster, a biosynthetic cluster of five genes was identified, which was highly homologous to the CLP brabantamide cluster described in *Pseudomonas* sp. SH-C52 (Schmidt et al., 2014; Van Der Voort et al., 2015). This biosynthetic cluster is also present in *P. fluorescens* In5 (Hennessy et al., 2017). BLAST analysis also revealed that 3 of the 21 genes differentially expressed were within the same open-reading frame (*ppsE_1*; *grsB_1*; *grsB_2*). These include all the three putative corpeptin NRPS genes (*tycB, dhbF_4, ppsE_1*; **Table 2**) and the two downstream genes coding for an ABC transporter system (*macA, macB2*; **Table 2** and **Figure 2A**). The genes *ppsE_1, macA*, and *macB* have been demonstrated to be part of the same transcriptional unit known as *crpCDE* (Strano et al., 2015). Insertional mutants in *crpC* and *crpD* were no longer able to produce corpeptins, but still produced cormycin (Strano et al., 2015). *CrpC* was significantly upregulated in the WT strain compared to the *pcoR* and *rfiA* mutants by 5.78 and 5.79 Log-fold changes (LogFC), respectively (**Table 2** and Supplementary Files 2, 3).

None of the putative cormycin NRPS genes were detected among the differentially expressed genes. However, two genes coding for an ABC transporter system and a gene annotated as *syrD_2* were over-expressed in the WT CFBP 5454 strain in comparison with *pcoR-* and *rfiA*-mutant strains (5.28- and 5.51-fold, respectively). In addition, among the differentially expressed genes, we identified the *yeaM* gene coding for an AraC family transcriptional regulator, in proximity of the putative cormycin NRPS genes, overexpressed 3.05- and 2.93-fold in WT compared to the *pcoR*- and *rfiA*-mutant strains, respectively. Four out five genes of the putative brabantamide biosynthesis cluster were differentially expressed. Although the production of this metabolite has not yet been described in *P. corrugata*, it could be argued that previous experimental conditions prevented it from being detected (Emanuele et al., 1998; Scaloni et al., 2004).

**FIGURE 3:**
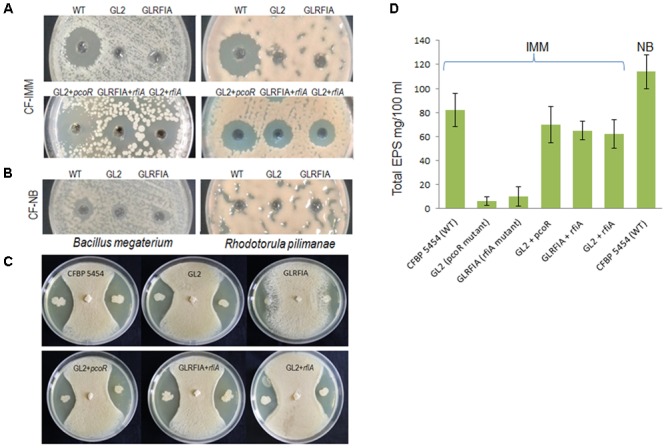
Mutational phenotypes of *P. corrugata* CFBP 5454 and derivatives mutants. **(A)** Antimicrobial activity of cell culture filtrates (10×) obtained in IMM (CF-IMM) of WT, GL2 (*pcoR* mutant), and GLRFIA (*rfiA* mutant) against CLP bioindicators *B. megaterium* and *R. pilimanae*. No activity was detected in *pcoR* and *rfiA* mutants. Complementation of *pcoR* (GL2+*pcoR*), *rfiA* (GLRFIA+*rfiA*), and of *rfiA* in the *pcoR* mutant (GL2+*rfiA*) restored antimicrobial activity. **(B)** Antimicrobial activity of cell culture filtrates (10×) obtained in NB (CF-NB). **(C)** Antimicrobial activity of bacterial cells of *P. corrugata* CFBP 5454, *pcoR*, and *rfiA* mutants and complemented mutants against *Penicillium digitatum*. **(D)** Total EPS produced after 4 days of incubation in IMM by the parent strain, *pcoR*, and *rfiA* mutants and complemented mutants and NB (only *P. corrugata* CFBP 5454).

Cell-free culture filtrates of the *pcoI*- and *rfiA*-mutant strains grown on IMM medium for all RNA extractions didn’t show antimicrobial activity against the two CLP bioindicators, the yeast *R. pilimanae* ATCC26423 and the Gram-positive bacterium *B. megaterium* ITM100 (**Figure 3A**) as opposed to the parent strain. The antagonistic activity was complemented at the same levels as those of the CFBP 5454 strain by expression *in trans* of the *pcoR* and *rfiA* genes into the respective mutant strains. In addition, the expression *in trans* of *rfiA* was sufficient to restore the antagonistic activity of the culture filtrate of the *pcoR* mutant (**Figure 3A**).

**Figure 3C** shows the antimicrobial activity of the living cells of strain CFBP 5454 and also the derivative mutants. It is worth noting that living cells of the two regulatory mutants still demonstrate antimicrobial activity against *P. digitatum* although to a different extent.

### Positive Regulation of the PcoR–RfiA Regulon on the Alginate Biosynthetic Cluster

RNA-seq analysis revealed that 12 genes putatively involved in the EPS alginate biosynthesis were upregulated in strain CFBP 5454 compared to both *luxR* derivative mutants with LogFC ranging from 4.09 (*algD*) to 1.93 (*algG*) in comparison with the *pcoR*-mutant strain, and from 4.09 (*algD*) to 2 (*algG*) to the *rfiA-*mutant strain (**Table 2** and Supplementary Files 2, 3).

In the *P. corrugata* CFBP 5454 genome, these genes were located in an 18 kb region constituting most of the core structural/biosynthetic cluster (contig38, **Figure 2B**) except for *algC* which was located elsewhere (contig86) and is not differentially expressed. This cluster encodes the biosynthetic enzymes and membrane-associated polymerization, modifications, and exports proteins necessary for the alginate production. The order and arrangement of the alginate structural gene cluster in *P. corrugata* is similar to those already described for *P. aeruginosa* and *P. syringae* (Fialho et al., 1990; Shankar et al., 1995; Peñaloza-Vázquez et al., 1997). The expression of other genes implicated in alginate regulation and switching phenotype and dispersed in other parts of the genome were not altered in these mutants (data not shown). No other EPS clusters in *Pseudomonas* strains, i.e., the *pel* and *psl* clusters described in *P. aeruginosa* (Franklin et al., 2011), the *epm* cluster, responsible for the production of an alginate-like EPS in *P. alkyphenolia* (Lee et al., 2014) or levane, were detected in the *P. corrugata* genome, as assessed by BLAST analysis. Total EPSs were isolated after isopropanol precipitation from the supernatant of the *pcoR* and *rfiA* mutant and complemented strains growing in IMM and compared to the *P. corrugata* parent strain CFBP 5454 (**Figure 3D**). An approximate 10-fold reduction of EPS yield was recorded in both mutants. The production of EPS was almost restored after complementation of the *pcoR* and *rfiA* (*P* ≤ 0.01) (**Figure 3D**). As *P. corrugata* has been demonstrated by Fett et al. (1996) to produce alginate as polymannuronic acid and not levan as EPSs, PcoR, and RfiA would seem to play a role in alginate production regulation.

### Other Genes Differentially Expressed in the Two *luxR* Mutants

In addition to the genes described above, there are eight genes whose expression was significantly modified in both the *pcoR* and *rfiA* mutants. Only two of them, *oprM_3* and *bepE_1*, are significantly down-expressed (LogFC ≥-2 and *P*-value ≤ 0.005) in the WT in comparison with mutant strains and are predicted to codify for multidrugs efflux systems. The *ompM_3* gene encodes a putative outer membrane protein and *bepE_1* an efflux pump membrane transporter (**Table 2**). Although they are located adjacently in the same genomic region, there was no evidence of their function. No gene homologs were found in the genome of the LMG 2172^T^. Three adjacently located genes (DIT1_1, *gph_2*, and DIT1_2) were over-expressed in the WT and showed more than a three-LogFC in transcript abundances compared to both *pcoR* and *rfiA* mutants. Analysis of the DIT1_2 putative protein revealed the presence of a DIT1_PvcA superfamily conserved domain, common to pyoverdine/dityrosine biosynthesis proteins. Blastx analysis showed a 40% homology with PvcA protein of *P. aeruginosa*, involved in the biosynthesis of the paerucumarin, a new metabolite described as an isonitrile functionalized cumarin (Clarke-Pearson and Brady, 2008). DIT1_1 differed from DIT1_2 in terms of an additional conserved domain belonging to the CAS-like superfamily, responsible for clavaminic acid biosynthesis. The *gph_2* gene encodes a putative phosphoglycolate phosphatase. Among the most differentially expressed genes (LogFC > 4 and *P*-value ≤ 0.005), *rhbA_1* also needs mentioning. This gene is a putative diaminobutyrate-2-oxoglutarate aminotransferase located in the ornicorrugatin gene cluster of *P. fluorescens* SBW25 and in histicorrugatin of *P. thivervalensis* (Cheng et al., 2013; Matthijs et al., 2016).

### Genes Regulated Independently by PcoR and RfiA

A total of 60 and 38 differentially expressed genes were identified in either the *pcoR*- or *rfiA-*mutant strains, respectively, mainly associated with transport systems, transcriptional regulation, and redox and oxidative stress. Of these, six transcriptional regulators were over-expressed in the WT in comparison to the *pcoR* mutant, although at low LogFC (0.77–1.10). Three of them belong to the HTH transcriptional regulator family, whose role to the best of our knowledge has not been investigated. Compared to the *rfiA* mutant in the WT strain, only one HTH regulator was over-expressed and two were down-expressed (LogFC 1) (Supplementary File 4). We observed that most of these genes had a very low LogFC < 1; thus, we decided to focus only genes with a minimum two LogFC. We found a strong overexpression of the *traI* gene coding for the AHL synthase (*pcoI* by Licciardello et al., 2007) in the WT compared to *pcoR* mutant. Without PcoR, the AHL-QS would not be able to work, since it is strictly dependent on the PcoR–AHL complex, and *pcoI* is only expressed at the basal level. Three genes involved in copper metabolism are among the most over-expressed in the WT compared to the *pcoR* mutant (Supplementary File 4). The Cyp4d2 gene, which codes for a cytochrome P450 involved in redox and oxidative stress, was differentially expressed only in the *pcoR* mutant. It is down-regulated in the WT with a LogFC of -2.4 (Supplementary Files 4, 5).

### Validation of the RNA-Seq Expression Patterns by Quantitative Real-Time PCR

**FIGURE 4:**
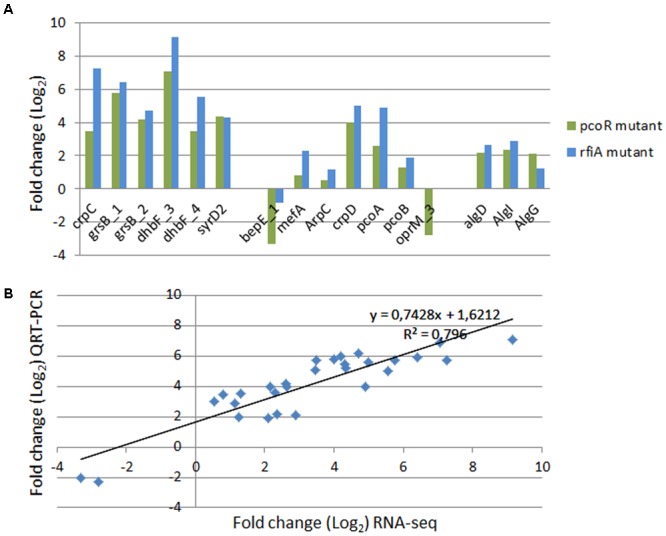
Validation by quantitative RT-PCR (qRT-PCR) of transcriptional patterns of randomly selected genes involved in CLPs and alginate production. **(A)** Transcriptional expression by qPCR in *P. corrugata* WT strain in comparison with GL2 and GLRFIA mutants grown on IMM for 40 h at the early stationary phase. The expression levels of all genes were standardized to the level of the constitutively expressed housekeeping 16S rDNA and normalized to expression in WT. The results represent the means of three independent experiments. **(B)** Correlation of estimates fold changes of differentially expressed transcripts between RNA-seq and qPCR analysis.

Thirteen genes among those co-regulated by PcoR and RfiA, putatively involved in biosynthesis secondary metabolites (six genes) and transport (seven genes), and three genes putatively responsible of alginate biosynthesis, were selected to validate RNA-seq results. qPCR was carried out with gene-specific primers (listed in Supplementary File 1) and the gene expression of WT versus the mutant strains was analyzed. Although there was a difference in the fold change estimated by the two methods (RNA-seq and qPCR), the expression pattern was the same (**Figure 4A**). A close correlation (Pearson’s *R*^2^ = 0.796) was observed between LogFC measured by RNA-seq and qPCR.

The data confirmed the positive regulation of PcoR and RfiA of all the selected genes, and the negative regulation of *bepE_1* and *opmR_3*, which were down-regulated in the WT compared to both mutants, in accordance with RNA-seq data.

### CLP and Alginate Gene Expression Analysis in Different Media and *in Planta* by qPCR

**FIGURE 5:**
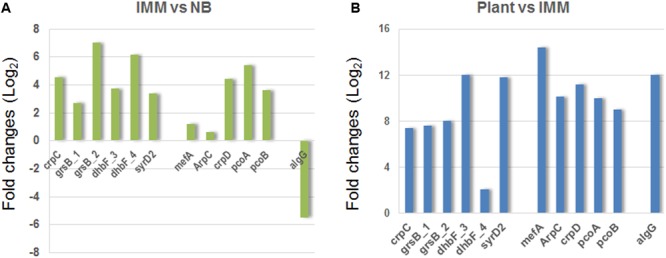
Relative expression of a subset of randomly selected genes from those already validated involved in CLPs and alginate production in *P. corrugata* CFBP 5454 grown in a rich medium, i.e., Nutrient broth (NB) **(A)** and in inoculated tomato plantlets **(B)** in comparison with growth in IMM by qPCR.

Since the RNA-seq experiment relied on conditions known to stimulate CLP production, we investigated the expression of NRP and alginate genes in the *P. corrugata* CFBP 5454 strain grown in complex undefined medium (NB) and *in planta.* The results demonstrated that genes involved in NRP biosynthesis and transport were activated two to sixfold more in minimal medium compared to NB, and two to five *in planta* compared to NB medium (**Figure 5**). Thus, cell culture filtrates of CFBP 5454 grown on NB showed very little or no activity against *B. megaterium* and *R. piliminae*, respectively (**Figure 3B**). *AlgG* gene in *P. corrugata* CFBP 5454 was upregulated both in NB and *in planta* compared to IMM. Total EPS production was higher in NB (114 ± 14 mg/100 ml) compared to IMM (82 ± 14 mg/100 ml), showing that rich medium provides better conditions for EPS production (**Figure 3D**).

## Discussion

In this study, we performed an *in vitro* transcriptome study to investigate the role of PcoR and RfiA LuxR-type transcriptional regulators in *P. corrugata* secondary metabolite production. Based on our previous results, we speculated that the two regulators play a pivotal role in the regulation of CLP biosynthetic loci, since cormycin and corpeptin production was impaired in *P. corrugata* CFBP 5454 derivative mutants (Licciardello et al., 2012). The overlapping of the RNA-seq data showed that approximately 50% of the genes cataloged in this study (approximately 3% of the annotated genes in the CFBP 5454 genome) were differentially expressed both in the *pcoR*- and *rfiA*-mutant strains compared to the *P. corrugata* CFBP 5454 parent strain. PcoR is the cognate receptor of AHLs, synthesized by PcoI in the *P. corrugata* QS system (Licciardello et al., 2007). In line to our previous results, the PcoR–AHL complex directly activates the transcriptional regulator gene *rfiA* since it is co-transcribed with *pcoI* (Licciardello et al., 2009). Thus, it is conceivable that this set of genes is co-regulated by both the LuxR-type transcriptional regulators.

Only 42 out of the 92 genes showed high log-fold-change values and were positively regulated, and only two genes were negatively regulated. Interestingly, almost all of the genes positively regulated are putatively involved in secondary metabolite production, namely genes involved in the biosynthesis of antimicrobial CLPs and in the production of alginic acid. As hypothesized, among these genes we found some whose involvement in the production of CLPs was clear and others whose involvement was likely.

Like many other biologically active secondary metabolites, CLPs are synthesized by multifunctional NRPSs (Raaijmakers et al., 2006). It is estimated that approximately 3 kb of DNA are required to code each amino acid activation module (Gross and Loper, 2009). Thus, due to the incomplete nature of the *P. corrugata* strain CFBP 5454 genome, the large CLP NRPs are divided into different contigs (Licciardello et al., 2014; Trantas et al., 2015). By conducting a BLAST analysis of genes down-regulated in *P. corrugata* CFBP 5454 *pcoR* and *rfiA* mutants and by genome mining the whole-genome sequence of strain LMG 2172^T^ in the GenBank repository, we ascertained that 19 differentially regulated genes were located in a large cluster which accounted for approximately 3.4% of the genome. This large DNA region includes putative gene clusters for cormycin and corpeptin and a brabantamide-like metabolite. This is consistent with the gene organization present in the biocontrol strain *Pseudomonas* SH-C52 for the thanapeptins, thanamycin, and brabantamide (Van Der Voort et al., 2015). In addition, *P. syringae* pv. *syringae* and *P. fluorescens* In5 produce both CLPs characterized by long peptide chains and smaller nonapeptides. Their biosynthesis clusters are adjacently located in the genome as in the case of syringopeptins and syringomycin (Scholz-Schroeder et al., 2001) and nunamycin and nunapeptins, respectively (Hennessy et al., 2017).

PcoR and RfiA regulate the genes for the three NRPSs necessary for the biosynthesis of corpeptins and the downstream located ABC transporter (*crpDE*). Some of these genes were described by Strano et al. (2015) who named them *crpCDE*, i.e., genes that are transcriptionally joined and which code for an NRPS and ABC efflux system. The introduction of a mutation in *crpC* yielded a *P. corrugata* strain, PCONRPS, which failed to produce corpeptins, thus demonstrating that *crpC* is part of the corpeptin biosynthesis locus. Gene disruption of *crpD* also affected the presence of corpeptins in the culture filtrates of *P. corrugata* CFBP 5454, supporting the assumption that CrpDE is the transport system involved in corpeptin export (Strano et al., 2015). Although the *pcoR* and *rfiA* mutants grown in the same conditions also failed to produce cormycin (Licciardello et al., 2012), we found none of the cormycin NRPS genes among the differentially expressed genes. Nevertheless, more genes that may be putatively involved in cormycin production were positively controlled by PcoR and RfiA. These included both a putative ABC transporter system, which is highly homologous to trasporters for the nonapeptides, syringomycin, and thanamycin in *P. syringae* B301D, B728A, and in *Pseudomonas* SHC52, and a gene annotated as *syrD*_2 coding an ATP-binding protein (Kang and Gross, 2005; Van Der Voort et al., 2015; Vaughn and Gross, 2016). *SyrD* flanks the corpeptin NRPS genes in *P. corrugata* and the syringopeptin gene cluster in *P. syringae* B301D. In the latter species *syrD* forms an operon with *sypA* and *sypB* NRPS genes; however, it is necessary for the secretion of both syringomycin and syringopeptin (Quigley et al., 1993; Wang et al., 2006a,b). In line with the possible similar organization of the transcriptional units, similar values of differential expression for putative *syrD, crpA*, and *crpB* (LogFC 5.19–5.30) were observed.

In previous works (Licciardello et al., 2007, 2009, 2012; Strano et al., 2015, 2017), we demonstrated that the expression of the cosmid pLC3.34 in *pcoR* mutant and of the plasmid pBBRRfia in *rfiA* mutant could complement the relative mutations, by restoring the virulence in tomato, the hypersensitivity response on *Nicotiana* spp., and the antimicrobial activity. The expression *in trans* of *rfiA* in the *pcoR* mutant was able to restore the virulence of the mutant at a similar level to the parent strain (Licciardello et al., 2009). We thus showed that the culture filtrates of the replicates of the mutant strains grown in IMM used for RNA-seq and qPCR were depleted in antimicrobial activity against CLP bioindicator strains. The activity is restored by complementation. Based on these results, RfiA is sufficient to also restore the investigated phenotypes in the *pcoR* mutant.

We previously demonstrated that the *pcoABC* operon, which is located in the CLP large cluster, is positive regulated by RfiA and, indirectly, by the PcoI/R system. RNA-seq data corroborated by qPCR validation data and phenotype complementation suggest that the regulation of *P. corrugata* genes in the PcoR–RfiA regulon may occur according to a hierarchical model. When a sufficient AHL signal has accumulated in the surrounding environment, it binds to PcoR and the complex upregulates *pcoI* gene in a positive feedback loop and consequently *rfiA*. RfiA, in turn, may activate the transcription of a number of genes either directly or indirectly. CLP biosynthesis clusters in *Pseudomonas* are flanked by multiple genes coding for LuxR transcriptional regulators (reviewed in Raaijmakers et al., 2010). Until recently the presence of a LuxR regulator directly linked to an AHL-QS system by gene cotranscription with acyl-homoserine lactone synthase gene has only been described for *P. corrugata, P. mediterranea*, and *Pseudomonas* sp. strain DF41 (Licciardello et al., 2009, 2012; Berry et al., 2014). Genes of the QS-RfiA system have been found to be conserved in *P. corrugata* and *P. mediterranea* (Trantas et al., 2015). This system is also conserved in other *P. corrugata, P. mediterranea* strains, and in *Pseudomonas* sp. SC-H52. *P. corrugata* CFBP 5454 PcoI, PcoR, and RfiA showed 100% protein homologies with the corresponding proteins in *P. corrugata* strain LMG 2172T, 85%, 95%, and 94% with strain *Pseudomonas* sp. SHC52, and of 84%, 95%, and 92% with *P. mediterranea* DSM16733T, respectively (data not shown).

The conservation of the QS system in this group of taxonomically related bacteria could have a biological significance. All of them have biocontrol properties mediated by the production of antimicrobial peptides. However, *P. corrugata* and *P. mediterranea* have been isolated as plant pathogens and are widespread pathogens in tomatoes (Catara, 2007). No type III secretion system or type III effectors are present in their genomes and the only relevant information regarding their interaction with plants is based on the pivotal role of QS and RfiA in virulence and in the hypersensitivity response in a non-host plant species and the putative/deduced role of CLPs in this interaction (Licciardello et al., 2009, 2014; Strano et al., 2015; Trantas et al., 2015). The transcriptomic data enlarge the number of secondary metabolites which are under the control of PcoR and RfiA in *P. corrugata* and for which a role *in planta* interaction needs to be further investigated. The high-density injection of *P. corrugata* cells in the stems of plant species belonging to different families led to pith necrosis. However, in nature the disease is widespread essentially in tomato (Siverio et al., 1993; Catara et al., 1997, 2002; Sutra et al., 1997; Catara, 2007). It is therefore conceivable that only in tomato can the bacterium reach the “quorum” cellular concentration required for the hierarchical activation of the genes under the QS control via RfiA including the metabolites with a phytotoxic activity that lead to the necrotrofic colonization of the plant resulting in TPN.

The role of plant signals in triggering the production of syringomycin and syringopeptins in the phytopathogen *P. syringae* pv. *syringae* via the sensor kinase GacS and the LuxR-type transcriptional regulators SalA and SyrF has been demonstrated (Mo and Gross, 1991; Wang et al., 2006a). As already reported for other *Pseudomonas*, the integration and networking of additional regulatory circuits may help *P. corrugata* to interact with environmental and metabolic signals in order to define the timing of the cell-based activation of QS (Fuqua et al., 1996; Venturi, 2006; Uzelac et al., 2017). In *Pseudomonas*, sp. strain DF41 AHL production and *pdfI* expression are under the positive control of the Gac/Rsm system (Berry et al., 2014).

Although CLPs produced by *P. corrugata* have a strong antimicrobial activity it seems that several other metabolites that are not regulated by PcorR and RfiA are involved in biocontrol activity. In fact, the cell-free culture filtrates of *PcoR* and *rfiA* mutants grown in IMM did not contain corpeptins and cormycin (Licciardello et al., 2012). In line with this result the two mutants did not have antimicrobial activity against the bioindicator strains even at 10-fold concentrations. The complementation of *rfiA* in the *pcoR* mutant is sufficient to restore antimicrobial activity thus demonstrating that at least its presence is necessary for the production of CLPs. Nevertheless, tests using bacterial cells suggest that more antimicrobial metabolites still have to be produced by the two mutant bacterial strains that are not regulated by PcoR and RfiA. Genome mining highlighted the presence of clusters for other metabolites, including the siderophore corrugatin, which could be involved in antimicrobial activity (Trantas et al., 2015). In addition, the role of volatile compounds in antagonistic activity has already been demonstrated (Trivedi et al., 2008; Strano et al., 2015).

PcoR and RfiA positively influence alginic acid biosynthesis gene expression. We found that almost all the genes belonging to the structural/biosynthetic cluster of the EPS alginate were upregulated in the WT compared to the *pcoR* and *rfiA* mutants. The production of alginate and not of levan has been demonstrated in a number of *P. corrugata* strains (Fett et al., 1996). Further characterization has shown that alginate consists solely of uronic acid (100% w/v) and mannose (Fett et al., 1996). Our analysis of the *P. corrugata* CFBP 5454 genome revealed that, similarly to other *Pseudomonas* spp. that belong to the rRNA homology group I (Fett et al., 1992), alginate biosynthesis and regulatory genes are widely distributed over three clusters namely, structural/biosynthetic, regulatory, and genetic switching genes (data not shown). A similar cluster has been found in the closely related species *P. mediterranea* (Licciardello et al., 2017) and *Pseudomonas* sp. SH-C52 genomes (Van Der Voort et al., 2015). Total EPSs were reduced in the two mutant strains analyzed in this study.

According to Fett et al. (1992, 1996) only alginate is produced by *P. corrugata* because there is no evidence of other gene clusters responsible for the synthesis of other EPS in other *Pseudomonas* species (Bradbury, 1986; Franklin et al., 2011). Alginate production is regulated by AHL in diverse *Pseudomonas*, including the phytopathogen *P. syringae* B728a in which it contributes to epiphytic fitness and resistance to desiccation, and increases resistance to oxidative stress (Quinones et al., 2005; Venturi, 2006). However, no significant effect of the *salA* mutation on alginate gene expression has been observed in *P. syringae* B301D (Wang et al., 2006b).

In addition, *P. corrugata* CFBP 5454 strain expression of some selected genes of the biosynthetic cluster for CLPs as investigated by qPCR was upregulated when the bacterium was grown in minimal medium compared to a rich complex undefined medium (NB). The opposite was observed for the expression of alginate gene *algG.* Genes coding for both secondary metabolites are overexpressed *in planta* compared to *in vitro* growth. These include genes for the biosynthesis of corpeptins, which are known to play a role in virulence in tomato (Strano et al., 2015).

Our results suggest that the QS-RfiA system in *P. corrugata* regulates hierarchically important secondary metabolites production at a high cell concentration. We focused on these metabolites since they play a pivotal role in the bacterial fitness of plant-associated bacteria in the interaction with other microorganisms as well as plants. RNA-seq generated a considerable amount of data, which merit future attention. However, it will be difficult to define the role of those genes for which differential values of expression are very low. Although their regulation is likely to depend on more intricate regulation networks influencing the fitness of the bacterium.

## Author Contributions

GL and VC conceived the study, and contributed to its design and coordination, and drafted the manuscript. AC contributed to the design and execution of RNA-seq data elaboration, performed and analyzed the RT-PCR experiments and phenotypic analysis. PB, CS, and AA contributed to mutants analysis by molecular and phenotypic assays. AC, PS, ET, GL, VC, NA, and RG contributed to the transcript data elaboration, genome comparison, and bioinformatics analysis. NA and RG contributed materials and bioinformatic tools. All authors contributed to the writing and editing of the manuscript and approved the final version of it.

## Conflict of Interest Statement

The authors declare that the research was conducted in the absence of any commercial or financial relationships that could be construed as a potential conflict of interest.

## Footnotes

1 https://img.jgi.doe.gov/

2 http://geneontology.org/
