# Supplementary material for: The LuxR Regulators PcoR and RfiA Co-regulate Antimicrobial Peptide and Alginate Production in Pseudomonas corrugata
Source: Front Microbiol. 2018 Mar 23;9:521. doi: 10.3389/fmicb.2018.00521 (PMC5890197; doi:10.3389/fmicb.2018.00521)
Supplement: Supplementary file 1 [file Table_1.DOCX]

Supplemental file 1. Oligonucleotides used to validate RNAseq results by qPCR

| **Gene** | **Sequences** | **T annealing °C** | **References** |
| --- | --- | --- | --- |
| mefA-fw | 5'-CAGTGTGTTTGCCTTGAT-3' | 59 | This study |
| mefA-rew | 5'-GAACCAATGATCGACCAG-3' | 59 | This study |
| ArpC-fw | 5'-CGCAAGACCTACAAGGAACA-3' | 59 | This study |
| ArpC-rew | 5'-CGCCAGCCTGTAGTAGTC-3' | 59 | This study |
| oprM_3-fw | 5'-CCATTCAGTACAAGGAAGG-3' | 56 | This study |
| oprM_3-rew | 5'-CATCTTCGGCATTGAGTC-3' | 56 | This study |
| bepE_1-fw | 5'-ATTCTCAACGTCAAGGAT-3' | 56 | This study |
| bepE_1-rew | 5'-AGCGAGTAATCACCAATA-3' | 56 | This study |
| grsB_1-fw | 5'-AGAGACCGTGCAGAACAT-3' | 59 | This study |
| grsB_1-rew | 5'-CGATGGATTGCCGTGAAT-3' | 59 | This study |
| grsB_2-fw | 5'-CTGAACTATCGTCACCTT-3' | 59 | This study |
| grsB_2-rew | 5'-CAGAACCTGGATACCTTC-3' | 59 | This study |
| dhbF_3-fw | 5'-CGTCAGTCGCCTATTGGA-3' | 59 | This study |
| dhbF_3-rew | 5'-GAAGCCGATGGAGTGGAA-3' | 59 | This study |
| dhbF_4-fw | 5'-TGAAACCGAAATCAATCTG-3' | 56 | This study |
| dhbF_4-rew | 5'-CGAAGAAATGATCGTGAC-3' | 56 | This study |
| AlgD-fw | 5'-CGAAAGCCCACTGGTAGAAC-3' | 58 | This study |
| AlgD-rew | 5'-AAGTCGGAGTTGAGCAAGGA-3' | 58 | This study |
| AlgI-fw | 5'-GGCTTCATCAAGAAAGTGTTC-3' | 58 | This study |
| AlgI-rew | 5'-ATGGACTGGCTGATGTAGGG-3' | 58 | This study |
| AlgG-fw | 5'-GTTCTCGGACATGTGGTACGG-3' | 58 | This study |
| AlgG-rew | 5'-GAAATGATGATCCCGTGCTT-3' | 58 | This study |
| syrD_2-fw | 5'-CAACTGAACACGACACTG-3' | 56 | This study |
| syrD_2-rew | 5'-GTAGGCAATACCGAACAG-3' | 56 | This study |
| Abc1 (CrpD)-fw | 5'-CAAAATCGCTATCGTGCTTGTC-3' | 60 | Strano et al., 2015 |
| Abc1 (CrpD)-rew | 5'-CGACCGTAGCGGTCAGGTA-3' | 60 | Strano et al., 2015 |
| Nrps (ppsE)-fw | 5'-ACGGGCCACCCGAAAG-3' | 60 | Strano et al., 2015 |
| Nrps (ppsE)-rew | 5'-GAGGCGAAAGCCACGTGAT-3' | 60 | Strano et al., 2015 |
| Pco16s-fw | 5'-TGTAGCGGTGAAATGCGTAGAT-3' | 58 | Conte et al., 2006 |
| Pco16s-rew | 5'-CCTCAGTGTCAGTATCAGTCCAG-3' | 58 | Conte et al., 2006 |
