# Supplementary material for: The LuxR Regulators PcoR and RfiA Co-regulate Antimicrobial Peptide and Alginate Production in Pseudomonas corrugata
Source: Front Microbiol. 2018 Mar 23;9:521. doi: 10.3389/fmicb.2018.00521 (PMC5890197; doi:10.3389/fmicb.2018.00521)
Supplement: Supplementary file 2 [file Table_2.DOCX]

| Supplemental file 2. Transcripts quantification in GL2 mutant of genes co-regulated by PcoR and RfiA | | | | | | | |
| --- | --- | --- | --- | --- | --- | --- | --- |
| **ID11** | **Contig** | **LogCPM** | **LogFC** | **P value** | **Gene product** | **Diff. expr.** | **GO** |
| oprM_3 | PCO_124 | 6.80 | -2.32 | 3.18E-34 | Outer membrane protein OprM | over GL2 | transporter activity |
| bepE_1 | PCO_124 | 7.91 | -2.06 | 2.24E-19 | Efflux pump membrane transporter BepE | over GL2 | transporter activity |
| fct_2 | PCO_121 | 7.88 | -1.71 | 2.40E-03 | Ferrichrysobactin receptor | over GL2 | transporter activity |
| HI_0362 | PCO_118 | 8.64 | -1.57 | 7.67E-04 | putative periplasmic iron-binding protein | over GL2 | membrane protein |
| ahpF | PCO_123 | 10.76 | -1.40 | 1.16E-05 | Alkyl hydroperoxide reductase subunit F | over GL2 | Redox and Oxidative stress |
| trxB_3 | PCO_119 | 6.26 | -1.35 | 5.04E-09 | Thioredoxin reductase | over GL2 | Redox and Oxidative stress |
| bepE_2 | PCO_35 | 10.33 | -1.32 | 8.19E-06 | Efflux pump membrane transporter BepE | over GL2 | transporter activity |
| mdtE | PCO_124 | 7.68 | -1.30 | 1.88E-08 | Multidrug resistance protein MdtE | over GL2 | transporter activity |
| ttgI | PCO_35 | 9.38 | -1.26 | 3.56E-05 | Toluene efflux pump outer membrane protein TtgI | over GL2 | transporter activity |
| bepF | PCO_35 | 10.42 | -1.21 | 4.62E-05 | Efflux pump periplasmic linker BepF | over GL2 | transporter activity |
| cat_1 | PCO_114 | 11.24 | -1.09 | 1.23E-02 | Catalase | over GL2 | transporter activity |
| katB | PCO_81 | 9.08 | -1.07 | 1.36E-03 | Catalase | over GL2 | Redox and Oxidative stress |
| ahpC | PCO_123 | 11.88 | -1.01 | 2.52E-03 | Alkyl hydroperoxide reductase subunit C | over GL2 | Redox and Oxidative stress |
| Alvin_1094 | PCO_81 | 6.34 | -0.99 | 4.79E-06 | hypothetical protein | over GL2 | unknown |
| yceJ_1 | PCO_109 | 6.89 | -0.92 | 1.32E-04 | hypothetical protein | over GL2 | unknown |
| qorA | PCO_112 | 10.15 | -0.91 | 7.93E-03 | Quinone oxidoreductase 1 | over GL2 | Redox and Oxidative stress |
| ydfG_1 | PCO_142 | 5.47 | -0.86 | 1.05E-04 | putative protein YdfG | over GL2 | unknown |
| mmgC_2 | PCO_101 | 11.01 | -0.82 | 3.51E-02 | Acyl-CoA dehydrogenase | over GL2 | fatty acid metabolism |
| fhaB_1 | PCO_106 | 7.90 | -0.81 | 7.84E-03 | Filamentous hemagglutinin | over GL2 | unknown |
| gutR | PCO_113 | 9.30 | -0.80 | 2.86E-02 | Transcription activator GutR | over GL2 | regulation of transcription. |
| eamB_3 | PCO_127 | 5.81 | -0.79 | 1.32E-04 | Cysteine/O-acetylserine efflux protein | over GL2 | transporter activity |
| ydeR | PCO_71 | 7.35 | -0.78 | 7.84E-03 | putative MFS-type transporter YdeR | over GL2 | unknown |
| alr_2 | PCO_72 | 7.38 | -0.77 | 2.79E-02 | Alanine racemase | over GL2 | aminoacid metabolism |
| PA3287 | PCO_71 | 7.26 | -0.74 | 1.58E-02 | Putative ankyrin repeat protein | over GL2 | unknown |
| yeiR | PCO_118 | 6.75 | -0.74 | 5.61E-03 | putative protein YeiR | over GL2 | others |
| tcmH | PCO_127 | 5.27 | -0.72 | 4.79E-02 | Tetracenomycin-F1 monooxygenase | over GL2 | Secondary metabolite production |
| hyuE | PCO_127 | 3.92 | -0.71 | 5.10E-03 | Hydantoin racemase | over GL2 | aminoacid metabolism |
| ywnA | PCO_119 | 5.28 | -0.71 | 9.51E-04 | Putative HTH-type transcriptional regulator YwnA | over GL2 | regulation of transcription. |
| fabR | PCO_103 | 7.10 | -0.64 | 3.51E-02 | HTH-type transcriptional repressor FabR | over GL2 | regulation of transcription. |
| DDB_G0269096 | PCO_153 | 4.85 | 0.55 | 3.61E-02 | Transmembrane protein | over WT | membrane protein |
| FI | PCO_115 | 4.73 | 0.58 | 2.45E-02 | Major tail sheath protein | over WT | others |
| ydcS | PCO_108 | 6.63 | 0.63 | 2.63E-02 | Putative ABC transporter periplasmic-binding protein YdcS | over WT | transporter activity |
| prr_2 | PCO_108 | 6.29 | 0.69 | 3.71E-03 | Gamma-aminobutyraldehyde dehydrogenase | over WT | Redox and Oxidative stress |
| N | PCO_115 | 4.97 | 0.70 | 8.67E-03 | Capsid proteins | over WT | unknown |
| SSU1 | PCO_96 | 6.26 | 0.74 | 1.90E-02 | Sulfite efflux pump SSU1 | over WT | transporter activity |
| RP587_1 | PCO_124 | 5.60 | 0.74 | 4.74E-02 | SCO2-like protein RP587 | over WT | transporter activity |
| cyc1 | PCO_124 | 5.10 | 0.84 | 8.25E-04 | Cytochrome c-552 | over WT | Redox and Oxidative stress |
| preA | PCO_90 | 7.27 | 0.93 | 1.80E-03 | NAD-dependent dihydropyrimidine dehydrogenase subunit PreA | over WT | Purine and pirimidine metabolism |
| Rv2030c_2 | PCO_112 | 6.33 | 0.96 | 1.09E-03 | putative proteinc/MT2089 | over WT | unknown |
| fabG_2 | PCO_124 | 6.11 | 1.00 | 1.79E-03 | 3-oxoacyl-[acyl-carrier-protein] reductase FabG | over WT | fatty acid metabolism |
| nemA_2 | PCO_124 | 6.79 | 1.03 | 6.12E-04 | N-ethylmaleimide reductase | over WT | Redox and Oxidative stress |
| nirC | PCO_105 | 4.99 | 1.08 | 1.11E-03 | Cytochrome c55X | over WT | Redox and Oxidative stress |
| MW2112 | PCO_107 | 5.54 | 1.09 | 1.05E-04 | Zinc-type alcohol dehydrogenase-like protein | over WT | Redox and Oxidative stress |
| NGR_a01370_2 | PCO_124 | 6.23 | 1.12 | 6.09E-05 | Putative aldehyde-dehydrogenase-like protein y4uC | over WT | fatty acid metabolism |
| aq_1546 | PCO_124 | 5.34 | 1.16 | 3.03E-04 | putative phosphosugar isomerase | over WT | carbohydrate metabolic process |
| pyd1 | PCO_90 | 7.58 | 1.19 | 2.73E-05 | Dihydropyrimidine dehydrogenase [NADP(+)] | over WT | Purine and pirimidine metabolism |
| dht | PCO_90 | 8.90 | 1.34 | 3.16E-05 | D-hydantoinase/dihydropyrimidinase | over WT | Purine and pirimidine metabolism |
| ybfB_1 | PCO_124 | 6.89 | 1.35 | 1.38E-06 | putative MFS-type transporter YbfB | over WT | others |
| pbuE | PCO_115 | 6.74 | 1.37 | 2.41E-06 | Purine efflux pump PbuE | over WT | transporter activity |
| cdhR_9 | PCO_124 | 7.52 | 1.51 | 2.41E-06 | HTH-type transcriptional regulator CdhR | over WT | regulation of transcription. |
| sstT | PCO_115 | 6.89 | 1.55 | 9.82E-08 | Serine/threonine transporter SstT | over WT | transporter activity |
| spvB_2 | PCO_36 | 7.78 | 1.79 | 5.98E-09 | Mono(ADP-ribosyl)transferase SpvB | over WT | Redox and Oxidative stress |
| algG | PCO_120 | 7.48 | 1.93 | 2.31E-04 | Poly(beta-D-mannuronate) C5 epimerase | over WT | alginic acid biosynthetic process |
| algE | PCO_120 | 7.56 | 1.95 | 1.80E-03 | Alginate production protein AlgE | over WT | alginic acid biosynthetic process |
| algI_1 | PCO_120 | 6.71 | 2.27 | 8.00E-06 | putative alginate O-acetylase AlgI | over WT | alginic acid biosynthetic process |
| algJ_1 | PCO_120 | 6.78 | 2.28 | 1.23E-06 | putative alginate O-acetylase AlgJ | over WT | alginic acid biosynthetic process |
| algL | PCO_120 | 7.07 | 2.29 | 2.20E-05 | Alginate lyase | over WT | alginic acid biosynthetic process |
| algX | PCO_120 | 7.13 | 2.33 | 4.59E-05 | Alginate biosynthesis protein AlgX | over WT | alginic acid biosynthetic process |
| nolG_1 | PCO_105 | 7.42 | 2.50 | 1.44E-18 | Nodulation protein NolG | over WT | transporter activity |
| algK | PCO_120 | 6.87 | 2.64 | 1.70E-05 | Alginate biosynthesis protein AlgK | over WT | alginic acid biosynthetic process |
| alg8 | PCO_120 | 7.44 | 2.70 | 9.07E-05 | Glycosyltransferase alg8 | over WT | alginic acid biosynthetic process |
| dltE_2 | PCO_124 | 6.86 | 2.85 | 1.93E-20 | putative oxidoreductase DltE | over WT | Redox and Oxidative stress |
| yeaM_3 | PCO_85 | 7.12 | 3.05 | 1.67E-16 | putative HTH-type transcriptional regulator YeaM | over WT | regulation of transcription. |
| algA | PCO_120 | 9.87 | 3.05 | 1.83E-06 | Alginate biosynthesis protein AlgA | over WT | alginic acid biosynthetic process |
| arpC | PCO_126 | 9.01 | 3.07 | 2.05E-11 | Antibiotic efflux pump outer membrane protein ArpC | over WT | membrane protein |
| alg44 | PCO_120 | 7.16 | 3.09 | 2.28E-05 | Alginate biosynthesis protein Alg44 | over WT | alginic acid biosynthetic process |
| gph_2 | PCO_88 | 6.25 | 3.15 | 3.81E-18 | Phosphoglycolate phosphatase | over WT | carbohydrate metabolic process |
| DIT1_1 | PCO_88 | 8.27 | 3.21 | 1.94E-15 | Spore wall maturation protein DIT1 | over WT | unknown |
| DIT1_2 | PCO_88 | 8.09 | 3.30 | 9.66E-14 | Spore wall maturation protein DIT1 | over WT | unknown |
| algF | PCO_120 | 7.89 | 3.38 | 9.41E-09 | Alginate biosynthesis protein AlgF | over WT | alginic acid biosynthetic process |
| mefA | PCO_85 | 6.94 | 3.49 | 2.11E-14 | Macrolide efflux protein A | over WT | transporter activity |
| nccB | PCO_105 | 5.77 | 3.52 | 3.13E-31 | Nickel-cobalt-cadmium resistance protein NccB | over WT | stress response |
| Hif1an | PCO_85 | 7.26 | 3.95 | 1.99E-13 | Hypoxia-inducible factor 1-alpha inhibitor | over WT | Redox and Oxidative stress |
| azoB_4 | PCO_124 | 8.50 | 4.05 | 1.93E-24 | NAD(P)H azoreductase | over WT | Redox and Oxidative stress |
| algD | PCO_120 | 10.12 | 4.09 | 2.50E-06 | GDP-mannose 6-dehydrogenase | over WT | alginic acid biosynthetic process |
| oprM_1 | PCO_105 | 6.90 | 4.39 | 2.27E-29 | Outer membrane protein OprM | over WT | transporter activity |
| rhbA_1 | PCO_105 | 7.94 | 4.42 | 2.22E-18 | Diaminobutyrate--2-oxoglutarate aminotransferase | over WT | Secondary metabolite production |
| hpxO | PCO_126 | 8.74 | 4.63 | 2.14E-26 | FAD-dependent urate hydroxylase | over WT | Purine and pirimidine metabolism |
| macB2 | PCO_105 | 9.30 | 4.71 | 7.55E-33 | Macrolide export ATP-binding/permease protein MacB 2 | over WT | transporter activity |
| dhbF_1 | PCO_126 | 10.80 | 4.85 | 1.91E-29 | Dimodular nonribosomal peptide synthase | over WT | Secondary metabolite production |
| pcaD_2 | PCO_64 | 9.53 | 4.89 | 5.40E-18 | 3-oxoadipate enol-lactonase 1 | over WT | Secondary metabolite production |
| yddQ_1 | PCO_114 | 9.61 | 4.92 | 2.79E-09 | putative isochorismatase family protein YddQ | over WT | Others |
| dhbF_4 | PCO_83 | 7.74 | 5.19 | 2.75E-58 | Dimodular nonribosomal peptide synthase | over WT | Secondary metabolite production |
| syrD_2 | PCO_64 | 9.36 | 5.28 | 5.50E-18 | ATP-binding protein SyrD | over WT | Secondary metabolite production |
| tycB | PCO_1 | 6.62 | 5.30 | 2.14E-66 | Tyrocidine synthase 2 | over WT | Secondary metabolite production |
| ATG26 | PCO_126 | 9.05 | 5.72 | 5.29E-21 | Sterol 3-beta-glucosyltransferase | over WT | transporter activity |
| grsB_1 | PCO_78 | 11.53 | 5.76 | 1.34E-47 | Gramicidin S synthase 2 | over WT | Secondary metabolite production |
| ppsE_1 | PCO_105 | 10.89 | 5.78 | 9.82E-59 | Plipastatin synthase subunit E | over WT | Secondary metabolite production |
| macA | PCO_105 | 8.96 | 5.85 | 2.47E-45 | Macrolide export protein MacA | over WT | transporter activity |
| grsB_2 | PCO_80 | 11.71 | 6.08 | 5.52E-54 | Gramicidin S synthase 2 | over WT | Secondary metabolite production |
| At3g21360_3 | PCO_64 | 9.12 | 6.15 | 2.34E-18 | Clavaminate synthase-like protein | over WT | Redox and Oxidative stress |
| dhbF_3 | PCO_64 | 11.24 | 6.99 | 6.28E-28 | Dimodular nonribosomal peptide synthase | over WT | Secondary metabolite production |
