# Supplementary material for: The LuxR Regulators PcoR and RfiA Co-regulate Antimicrobial Peptide and Alginate Production in Pseudomonas corrugata
Source: Front Microbiol. 2018 Mar 23;9:521. doi: 10.3389/fmicb.2018.00521 (PMC5890197; doi:10.3389/fmicb.2018.00521)
Supplement: Supplementary file 3 [file Table_3.DOCX]

| Supplemental file 3. Transcripts quantification in GLRFIA mutant of genes co-regulated by PcoR and RfiA | | | | | | | |
| --- | --- | --- | --- | --- | --- | --- | --- |
| **ID** | **Contig** | **LogCPM** | **LogFC** | **P value** | **Gene product** | **Diff expres** | **GO** |
| oprM_3 | PCO_124 | 6.83 | -2.13 | 0.00060 | Outer membrane protein OprM | over GLRFIA | transporter activity |
| bepE_1 | PCO_124 | 8.05 | -2.03 | 0.00082 | Efflux pump membrane transporter BepE | over GLRFIA | transporter activity |
| ahpF | PCO_123 | 11.22 | -1.85 | 0.00000 | Alkyl hydroperoxide reductase subunit F | over GLRFIA | Redox and Oxidative stress |
| cat_1 | PCO_114 | 11.72 | -1.63 | 0.00000 | Catalase | over GLRFIA | transporter activity |
| mdtE | PCO_124 | 8.01 | -1.61 | 0.00000 | Multidrug resistance protein MdtE | over GLRFIA | transporter activity |
| fct_2 | PCO_121 | 7.83 | -1.46 | 0.03545 | Ferrichrysobactin receptor | over GLRFIA | transporter activity |
| HI_0362 | PCO_118 | 8.65 | -1.40 | 0.01480 | putative periplasmic iron-binding protein | over GLRFIA | membrane protein |
| ahpC | PCO_123 | 12.18 | -1.33 | 0.00000 | Alkyl hydroperoxide reductase subunit C | over GLRFIA | Redox and Oxidative stress |
| trxB_3 | PCO_119 | 6.34 | -1.30 | 0.00225 | Thioredoxin reductase | over GLRFIA | Redox and Oxidative stress |
| katB | PCO_81 | 9.24 | -1.18 | 0.00045 | Catalase | over GLRFIA | Redox and Oxidative stress |
| Alvin_1094 | PCO_81 | 6.50 | -1.12 | 0.00083 | hypothetical protein | over GLRFIA | unknown |
| bepF | PCO_35 | 10.45 | -1.12 | 0.00010 | Efflux pump periplasmic linker BepF | over GLRFIA | transporter activity |
| bepE_2 | PCO_35 | 10.26 | -1.06 | 0.00366 | Efflux pump membrane transporter BepE | over GLRFIA | transporter activity |
| PA3287 | PCO_71 | 7.51 | -1.04 | 0.00085 | Putative ankyrin repeat protein | over GLRFIA | unknown |
| ttgI | PCO_35 | 9.32 | -1.02 | 0.00528 | Toluene efflux pump outer membrane protein TtgI | over GLRFIA | transporter activity |
| eamB_3 | PCO_127 | 5.98 | -0.97 | 0.00000 | Cysteine/O-acetylserine efflux protein | over GLRFIA | transporter activity |
| mmgC_2 | PCO_101 | 11.16 | -0.96 | 0.00298 | Acyl-CoA dehydrogenase | over GLRFIA | fatty acid metabolism |
| qorA | PCO_112 | 10.25 | -0.95 | 0.00221 | Quinone oxidoreductase 1 | over GLRFIA | Redox and Oxidative stress |
| alr_2 | PCO_72 | 7.55 | -0.94 | 0.00543 | Alanine racemase | over GLRFIA | transporter activity |
| tcmH | PCO_127 | 5.42 | -0.88 | 0.00554 | Tetracenomycin-F1 monooxygenase | over GLRFIA | Secondary metabolite production |
| gutR | PCO_113 | 9.35 | -0.80 | 0.03499 | Transcription activator GutR | over GLRFIA | regulation of transcription. |
| yceJ_1 | PCO_109 | 6.88 | -0.79 | 0.00187 | hypothetical protein | over GLRFIA | unknown |
| fhaB_1 | PCO_106 | 7.96 | -0.79 | 0.04930 | Filamentous hemagglutinin | over GLRFIA | unknown |
| ydeR | PCO_71 | 7.41 | -0.77 | 0.00586 | putative MFS-type transporter YdeR | over GLRFIA | transporter activity |
| fabR | PCO_103 | 7.21 | -0.74 | 0.02078 | HTH-type transcriptional repressor FabR | over GLRFIA | regulation of transcription. |
| ywnA | PCO_119 | 5.36 | -0.74 | 0.00278 | Putative HTH-type transcriptional regulator YwnA | over GLRFIA | regulation of transcription. |
| hyuE | PCO_127 | 3.99 | -0.74 | 0.02595 | Hydantoin racemase | over GLRFIA | aminoacid metabolism |
| yeiR | PCO_118 | 6.77 | -0.69 | 0.01573 | putative protein YeiR | over GLRFIA | Others |
| ydfG_1 | PCO_142 | 5.36 | -0.58 | 0.03696 | putative protein YdfG | over GLRFIA | Others |
| prr_2 | PCO_108 | 6.24 | 0.59 | 0.03424 | Gamma-aminobutyraldehyde dehydrogenase | over WT | Redox and Oxidative stress |
| ydcS | PCO_108 | 6.57 | 0.59 | 0.04552 | Putative ABC transporter periplasmic-binding protein YdcS | over WT | transporter activity |
| DDB_G0269096 | PCO_153 | 4.73 | 0.68 | 0.00271 | Transmembrane protein | over WT | membrane protein |
| fabG_2 | PCO_124 | 6.10 | 0.72 | 0.03424 | 3-oxoacyl-[acyl-carrier-protein] reductase FabG | over WT | fatty acid metabolism metabolism |
| cyc1 | PCO_124 | 5.04 | 0.72 | 0.00199 | Cytochrome c-552 | over WT | Redox and Oxidative stress |
| FI | PCO_115 | 4.60 | 0.74 | 0.00132 | Major tail sheath protein | over WT | Others |
| Rv2030c_2 | PCO_112 | 6.28 | 0.78 | 0.03019 | putative proteinc/MT2089 | over WT | unknown |
| MW2112 | PCO_107 | 5.52 | 0.79 | 0.00333 | Zinc-type alcohol dehydrogenase-like protein | over WT | Redox and Oxidative stress |
| SSU1 | PCO_96 | 6.15 | 0.79 | 0.01210 | Sulfite efflux pump SSU1 | over WT | transporter activity |
| nirC | PCO_105 | 4.97 | 0.80 | 0.02559 | Cytochrome c55X | over WT | Redox and Oxidative stress |
| N | PCO_115 | 4.84 | 0.81 | 0.00011 | Capsid proteins | over WT | unknown |
| RP587_1 | PCO_124 | 5.44 | 0.95 | 0.00045 | SCO2-like protein RP587 | over WT | transporter activity |
| spvB_2 | PCO_36 | 7.79 | 1.11 | 0.00003 | Mono(ADP-ribosyl)transferase SpvB | over WT | Redox and Oxidative stress |
| nemA_2 | PCO_124 | 6.65 | 1.11 | 0.00006 | N-ethylmaleimide reductase | over WT | Redox and Oxidative stress |
| aq_1546 | PCO_124 | 5.21 | 1.20 | 0.00001 | putative phosphosugar isomerase | over WT | carbohydrate metabolic process |
| preA | PCO_90 | 7.07 | 1.23 | 0.00000 | NAD-dependent dihydropyrimidine dehydrogenase subunit PreA | over WT | Purine and pirimidine metabolism |
| NGR_a01370_2 | PCO_124 | 6.07 | 1.26 | 0.00000 | Putative aldehyde-dehydrogenase-like protein y4uC | over WT | fatty acid metabolism |
| pbuE | PCO_115 | 6.62 | 1.29 | 0.00000 | Purine efflux pump PbuE | over WT | transporter activity |
| Dht | PCO_90 | 8.78 | 1.29 | 0.00002 | D-hydantoinase/dihydropyrimidinase | over WT | Purine and pirimidine metabolism |
| ybfB_1 | PCO_124 | 6.74 | 1.37 | 0.00000 | putative MFS-type transporter YbfB | over WT | Others |
| cdhR_9 | PCO_124 | 7.39 | 1.47 | 0.00000 | HTH-type transcriptional regulator CdhR | over WT | regulation of transcription. |
| pyd1 | PCO_90 | 7.37 | 1.49 | 0.00000 | Dihydropyrimidine dehydrogenase [NADP(+)] | over WT | Purine and pirimidine metabolism |
| sstT | PCO_115 | 6.74 | 1.51 | 0.00000 | Serine/threonine transporter SstT | over WT | transporter activity |
| algG | PCO_120 | 7.28 | 2.00 | 0.00000 | Poly(beta-D-mannuronate) C5 epimerase | over WT | alginic acid biosynthetic process |
| algI_1 | PCO_120 | 6.53 | 2.15 | 0.00000 | putative alginate O-acetylase AlgI | over WT | alginic acid biosynthetic process |
| algE | PCO_120 | 7.33 | 2.22 | 0.00000 | Alginate production protein AlgE | over WT | alginic acid biosynthetic process |
| algX | PCO_120 | 6.91 | 2.42 | 0.00000 | Alginate biosynthesis protein AlgX | over WT | alginic acid biosynthetic process |
| algJ_1 | PCO_120 | 6.55 | 2.43 | 0.00000 | putative alginate O-acetylase AlgJ | over WT | alginic acid biosynthetic process |
| algL | PCO_120 | 6.84 | 2.45 | 0.00000 | Alginate lyase | over WT | alginic acid biosynthetic process |
| algK | PCO_120 | 6.66 | 2.56 | 0.00000 | Alginate biosynthesis protein AlgK | over WT | alginic acid biosynthetic process |
| nolG_1 | PCO_105 | 7.19 | 2.59 | 0.00000 | Nodulation protein NolG | over WT | transporter activity |
| dltE_2 | PCO_124 | 6.66 | 2.71 | 0.00000 | putative oxidoreductase DltE | over WT | Redox and Oxidative stress |
| alg8 | PCO_120 | 7.21 | 2.75 | 0.00000 | Glycosyltransferase alg8 | over WT | alginic acid biosynthetic process |
| arpC | PCO_126 | 8.79 | 2.92 | 0.00000 | Antibiotic efflux pump outer membrane protein ArpC | over WT | membrane protein |
| yeaM_3 | PCO_85 | 6.90 | 2.93 | 0.00000 | putative HTH-type transcriptional regulator YeaM | over WT | regulation of transcription. |
| algA | PCO_120 | 9.65 | 2.98 | 0.00000 | Alginate biosynthesis protein AlgA | over WT | alginic acid biosynthetic process |
| algF | PCO_120 | 7.65 | 3.28 | 0.00000 | Alginate biosynthesis protein AlgF | over WT | alginic acid biosynthetic process |
| alg44 | PCO_120 | 6.90 | 3.30 | 0.00000 | Alginate biosynthesis protein Alg44 | over WT | alginic acid biosynthetic process |
| gph_2 | PCO_88 | 5.99 | 3.34 | 0.00000 | Phosphoglycolate phosphatase | over WT | carbohydrate metabolic process |
| DIT1_2 | PCO_88 | 7.83 | 3.52 | 0.00000 | Spore wall maturation protein DIT1 | over WT | unknown |
| DIT1_1 | PCO_88 | 8.00 | 3.57 | 0.00000 | Spore wall maturation protein DIT1 | over WT | unknown |
| mefA | PCO_85 | 6.67 | 3.68 | 0.00000 | Macrolide efflux protein A | over WT | transporter activity |
| nccB | PCO_105 | 5.51 | 3.83 | 0.00000 | Nickel-cobalt-cadmium resistance protein NccB | over WT | stress response |
| Hif1an | PCO_85 | 7.00 | 4.01 | 0.00000 | Hypoxia-inducible factor 1-alpha inhibitor | over WT | Redox and Oxidative stress |
| algD | PCO_120 | 9.86 | 4.09 | 0.00000 | GDP-mannose 6-dehydrogenase | over WT | alginic acid biosynthetic process |
| azoB_4 | PCO_124 | 8.24 | 4.23 | 0.00000 | NAD(P)H azoreductase | over WT | Redox and Oxidative stress |
| oprM_1 | PCO_105 | 6.64 | 4.29 | 0.00000 | Outer membrane protein OprM | over WT | transporter activity |
| hpxO | PCO_126 | 8.47 | 4.69 | 0.00000 | FAD-dependent urate hydroxylase | over WT | Purine and pirimidine metabolism |
| rhbA_1 | PCO_105 | 7.66 | 4.81 | 0.00000 | Diaminobutyrate--2-oxoglutarate aminotransferase | over WT | Secondary metabolite 1production |
| macB2 | PCO_105 | 9.03 | 4.83 | 0.00000 | Macrolide export ATP-binding/permease protein MacB 2 | over WT | transporter activity |
| dhbF_1 | PCO_126 | 10.53 | 4.84 | 0.00000 | Dimodular nonribosomal peptide synthase | over WT | Secondary metabolite production |
| pcaD_2 | PCO_64 | 9.26 | 4.94 | 0.00000 | 3-oxoadipate enol-lactonase 1 | over WT | Secondary metabolite production |
| dhbF_4 | PCO_83 | 7.48 | 5.02 | 0.00000 | Dimodular nonribosomal peptide synthase | over WT | Secondary metabolite production |
| yddQ_1 | PCO_114 | 9.33 | 5.09 | 0.00000 | putative isochorismatase family protein YddQ | over WT | Others |
| tycB | PCO_1 | 6.34 | 5.42 | 0.00000 | Tyrocidine synthase 2 | over WT | Secondary metabolite production |
| syrD_2 | PCO_64 | 9.08 | 5.51 | 0.00000 | ATP-binding protein SyrD | over WT | Secondary metabolite production |
| macA | PCO_105 | 8.68 | 5.69 | 0.00000 | Macrolide export protein MacA | over WT | transporter activity |
| ATG26 | PCO_126 | 8.77 | 5.72 | 0.00000 | Sterol 3-beta-glucosyltransferase | over WT | transporter activity |
| ppsE_1 | PCO_105 | 10.61 | 5.79 | 0.00000 | Plipastatin synthase subunit E | over WT | Secondary metabolite production |
| grsB_1 | PCO_78 | 11.25 | 5.95 | 0.00000 | Gramicidin S synthase 2 | over WT | Secondary metabolite production |
| grsB_2 | PCO_80 | 11.43 | 6.25 | 0.00000 | Gramicidin S synthase 2 | over WT | Secondary metabolite production |
| At3g21360_3 | PCO_64 | 8.83 | 6.55 | 0.00000 | Clavaminate synthase-like protein | over WT | Redox and Oxidative stress |
| dhbF_3 | PCO_64 | 10.95 | 7.18 | 0.00000 | Dimodular nonribosomal peptide synthase | over WT | Secondary metabolite production |
