# Supplementary material for: The LuxR Regulators PcoR and RfiA Co-regulate Antimicrobial Peptide and Alginate Production in Pseudomonas corrugata
Source: Front Microbiol. 2018 Mar 23;9:521. doi: 10.3389/fmicb.2018.00521 (PMC5890197; doi:10.3389/fmicb.2018.00521)
Supplement: Supplementary file 4 [file Table_4.DOCX]

| Supplemental file 4. Transcripts quantification genes differentially expressed only in GL2 mutant | | | | | | | |
| --- | --- | --- | --- | --- | --- | --- | --- |
| **ID** | **contig** | **LogCPM** | **LogFC** | **PVALUE** | **gene_product** | **Diff.expr.** | **GO** |
| HI_1624 | PCO_121 | 7.92 | -2.06 | 0.01722 | putative protein | over GL2 | unknown |
| TM_0124 | PCO_118 | 8.12 | -1.94 | 0.02598 | putative metal transport system ATP-binding protein | over GL2 | transporter activity |
| folE2_1 | PCO_118 | 9.15 | -1.91 | 0.02454 | GTP cyclohydrolase FolE2 | over GL2 | unknown |
| yjgH | PCO_102 | 4.12 | -1.56 | 0.00000 | RutC family protein YjgH | over GL2 | unknown |
| CBSX3_1 | PCO_119 | 5.26 | -1.07 | 0.02786 | CBS domain-containing protein CBSX3%2C mitochondrial | over GL2 | unknown |
| MJ0761 | PCO_26 | 7.74 | -0.96 | 0.00380 | putative deoxyribonuclease | over GL2 | unknown |
| yjcH | PCO_71 | 7.73 | -0.94 | 0.00180 | Inner membrane protein YjcH | over GL2 | unknown |
| fusA_3 | PCO_70 | 7.29 | -0.90 | 0.00019 | Fusaric acid resistance protein FusA | over GL2 | unknown |
| actP_2 | PCO_71 | 9.02 | -0.89 | 0.01447 | Cation/acetate symporter ActP | over GL2 | Redox and Oxidative stress |
| ygaZ | PCO_127 | 5.06 | -0.87 | 0.00006 | Inner membrane protein YgaZ | over GL2 | transporter activity |
| HI_1511 | PCO_88 | 8.68 | -0.85 | 0.03903 | Mu-like prophage FluMu tail sheath protein | over GL2 | unknown |
| aroB_1 | PCO_26 | 8.22 | -0.74 | 0.03834 | 3-dehydroquinate synthase | over GL2 | aminoacid metabolism |
| hpmB_2 | PCO_24 | 5.63 | -0.69 | 0.00308 | Hemolysin transporter protein HpmB | over GL2 | transporter activity |
| HI_1008 | PCO_110 | 6.89 | -0.67 | 0.01071 | putative protein | over GL2 | unknown |
| plu0081 | PCO_154 | 5.16 | -0.67 | 0.00407 | hypothetical protein | over GL2 | unknown |
| ykwD_2 | PCO_119 | 6.20 | -0.55 | 0.02454 | putative protein YkwD | over GL2 | others |
| pucI_2 | PCO_127 | 5.39 | -0.55 | 0.02897 | putative allantoin permease | over GL2 | transporter activity |
| aaeB | PCO_85 | 5.28 | 0.61 | 0.03512 | p-hydroxybenzoic acid efflux pump subunit AaeB | over WT | transporter activity |
| Acad10 | PCO_95 | 6.57 | 0.65 | 0.03512 | Acyl-CoA dehydrogenase family member 10 | over WT | Redox and Oxidative stress |
| HMT3 | PCO_118 | 7.31 | 0.66 | 0.04219 | Homocysteine S-methyltransferase 3 | over WT | aminoacid metabolism |
| osmY_3 | PCO_124 | 6.07 | 0.67 | 0.04743 | Osmotically-inducible protein Y | over WT | transporter activity |
| M | PCO_115 | 3.33 | 0.68 | 0.04984 | Terminase%2C endonuclease subunit | over WT | others |
| uspE_1 | PCO_109 | 6.33 | 0.70 | 0.04504 | hypothetical protein | over WT | stress protein |
| groL5 | PCO_124 | 6.72 | 0.72 | 0.03811 | 60 kDa chaperonin 5 | over WT | stress protein |
| yhbT | PCO_140 | 5.50 | 0.74 | 0.04701 | putative protein YhbT | over WT | transporter activity |
| greB_2 | PCO_56 | 5.92 | 0.77 | 0.01126 | Transcription elongation factor GreB | over WT | regulation of transcription. |
| uspE_6 | PCO_124 | 5.51 | 0.79 | 0.00510 | Universal stress protein E | over WT | stress protein |
| AF_1420 | PCO_117 | 7.68 | 0.79 | 0.03696 | putative protein | over WT | unknown |
| pdhC | PCO_112 | 5.82 | 0.82 | 0.01758 | Dihydrolipoyllysine-residue acetyltrans component of pyr dehydr complex | over WT | carbohydrate metabolic process |
| DR_1438 | PCO_112 | 5.22 | 0.86 | 0.00509 | putative ABC transporter-binding protein | over WT | transporter activity |
| PA4778 | PCO_107 | 5.24 | 0.86 | 0.00041 | putative HTH-type transcriptional regulator | over WT | regulation of transcription. |
| osmY_2 | PCO_112 | 6.90 | 0.87 | 0.01369 | Osmotically-inducible protein Y | over WT | stress protein |
| hcnA_3 | PCO_90 | 7.71 | 0.88 | 0.04198 | Hydrogen cyanide synthase subunit HcnA | over WT | Secondary metabolite production |
| adhA | PCO_109 | 6.63 | 0.88 | 0.02170 | putative alcohol dehydrogenase AdhA | over WT | Redox and Oxidative stress |
| osmY_1 | PCO_109 | 7.23 | 0.88 | 0.01018 | Osmotically-inducible protein Y | over WT | transporter activity |
| Rv0571c_1 | PCO_112 | 5.52 | 0.89 | 0.02061 | Putative phosphoribosyl transferasec/MT0597 | over WT | others |
| trpI_2 | PCO_156 | 6.72 | 0.90 | 0.00022 | HTH-type transcriptional regulator TrpI | over WT | regulation of transcription. |
| yhbU | PCO_140 | 7.03 | 0.91 | 0.03270 | putative protease YhbU | over WT | others |
| cdhR_10 | PCO_124 | 7.41 | 0.91 | 0.00856 | HTH-type transcriptional regulator CdhR | over WT | regulation of transcription. |
| uspE_2 | PCO_109 | 7.11 | 0.92 | 0.01167 | Universal stress protein E | over WT | stress protein |
| RPA1673 | PCO_109 | 7.98 | 0.93 | 0.03040 | putative phosphoketolase | over WT | carbohydrate metabolic process |
| bkdA1 | PCO_123 | 7.81 | 0.93 | 0.01976 | 2-oxoisovalerate dehydrogenase subunit alpha | over WT | Redox and Oxidative stress |
| yfiQ | PCO_109 | 7.39 | 0.94 | 0.00404 | putative protein YfiQ | over WT | unknown |
| Rv2030c_1 | PCO_112 | 7.56 | 0.97 | 0.01758 | putative proteinc/MT2089 | over WT | unknown |
| pdxH_1 | PCO_140 | 7.95 | 0.99 | 0.03354 | Pyridoxine/pyridoxamine 5'-phosphate oxidase | over WT | Redox and Oxidative stress |
| yhiI | PCO_109 | 7.32 | 1.00 | 0.01758 | putative protein YhiI | over WT | transporter activity |
| uspE_5 | PCO_112 | 7.02 | 1.03 | 0.00083 | Universal stress protein E | over WT | unknown |
| hemN_2 | PCO_126 | 9.31 | 1.05 | 0.00784 | Oxygen-independent coproporphyrinogen-III oxidase | over WT | Redox and Oxidative stress |
| arcD | PCO_109 | 8.95 | 1.05 | 0.01409 | Arginine/ornithine antiporter | over WT | transporter activity |
| nirM | PCO_105 | 7.70 | 1.05 | 0.00793 | Cytochrome c-551 | over WT | Redox and Oxidative stress |
| pyrR_2 | PCO_112 | 6.20 | 1.08 | 0.00252 | Bifunctional protein PyrR | over WT | regulation of transcription. |
| echR | PCO_105 | 7.28 | 1.10 | 0.00013 | Transcriptional activator protein EchR | over WT | regulation of transcription. |
| uspE_4 | PCO_109 | 9.47 | 1.13 | 0.01443 | Universal stress protein E | over WT | stress protein |
| bkdA2 | PCO_123 | 6.78 | 1.19 | 0.00057 | 2-oxoisovalerate dehydrogenase subunit beta | over WT | unknow |
| hemN_3 | PCO_126 | 9.45 | 1.23 | 0.00896 | Oxygen-independent coproporphyrinogen-III oxidase | over WT | Redox and Oxidative stress |
| map_2 | PCO_85 | 6.79 | 1.24 | 0.00106 | Methionine aminopeptidase | over WT | aminoacid metabolism |
| copA_2 | PCO_107 | 7.82 | 1.52 | 0.00000 | Copper-exporting P-type ATPase A | over WT | transporter activity |
| copZ_1 | PCO_107 | 4.88 | 2.01 | 0.00000 | Copper chaperone CopZ | over WT | transporter activity |
| copZ_2 | PCO_156 | 6.18 | 2.27 | 0.00000 | Copper chaperone CopZ | over WT | transporter activity |
| traI | PCO_105 | 9.32 | 5.60 | 0.00000 | Acyl-homoserine-lactone synthase | over WT | quorum sensing |
