# Supplementary material for: The LuxR Regulators PcoR and RfiA Co-regulate Antimicrobial Peptide and Alginate Production in Pseudomonas corrugata
Source: Front Microbiol. 2018 Mar 23;9:521. doi: 10.3389/fmicb.2018.00521 (PMC5890197; doi:10.3389/fmicb.2018.00521)
Supplement: Supplementary file 5 [file Table_5.DOCX]

| Supplemental file 5. Transcripts quantification genes differentially expressed only in GLRFIA mutant | | | | | | | |
| --- | --- | --- | --- | --- | --- | --- | --- |
| **ID** | **contig** | **LogCPM** | **LogFC** | **P value** | **Gene_product** | **Diff. Expr.** | **GO** |
| Cyp4d2 | PCO_116 | 6.92 | -2.49 | 0.04552 | Cytochrome P450 4d2 | over GLRFIA | Redox and Oxidative stress |
| PA4923 | PCO_103 | 7.54 | -1.00 | 0.01002 | LOG family protein | over GLRFIA | Others |
| aq_740 | PCO_148 | 8.88 | -0.96 | 0.00068 | hypothetical protein | over GLRFIA | transporter activity |
| GPX2 | PCO_128 | 7.75 | -0.96 | 0.00119 | Glutathione peroxidase 2 | over GLRFIA | Redox and Oxidative stress |
| yadG | PCO_127 | 9.41 | -0.91 | 0.00390 | putative ABC transporter ATP-binding protein YadG | over GLRFIA | transporter activity |
| algE3 | PCO_112 | 5.74 | -0.86 | 0.00017 | Poly(beta-D-mannuronate) C5 epimerase 3 | over GLRFIA | alginic acid biosynthetic process |
| mtcA1 | PCO_124 | 11.24 | -0.86 | 0.00543 | Beta-carbonic anhydrase 1 | over GLRFIA | Others |
| ribA | PCO_114 | 8.36 | -0.85 | 0.00543 | GTP cyclohydrolase-2 | over GLRFIA | Others |
| Rv0196 | PCO_124 | 6.73 | -0.81 | 0.00294 | putative HTH-type transcriptional regulator/MT0206 | over GLRFIA | regulation of transcription. |
| yadH | PCO_127 | 8.51 | -0.77 | 0.02479 | Inner membrane transport permease YadH | over GLRFIA | membrane protein |
| ohrR_2 | PCO_128 | 6.46 | -0.76 | 0.00826 | Organic hydroperoxide resistance transcriptional regulator | over GLRFIA | regulation of transcription. |
| HD_0322 | PCO_72 | 7.41 | -0.75 | 0.03696 | RutC family protein | over GLRFIA | Purine and pirimidine metabolism |
| Mb2924c | PCO_117 | 11.02 | -0.72 | 0.03361 | putative oxidoreductasec | over GLRFIA | Redox and Oxidative stress |
| fdhD | PCO_117 | 9.26 | -0.72 | 0.02866 | hypothetical protein | over GLRFIA | hypothetical protein |
| yaiW | PCO_47 | 6.51 | -0.70 | 0.01488 | putative protein YaiW | over GLRFIA | Others |
| yfdC | PCO_109 | 6.24 | -0.69 | 0.03361 | Inner membrane protein YfdC | over GLRFIA | membrane protein |
| modB | PCO_123 | 5.75 | -0.68 | 0.00275 | Molybdenum transport system permease protein ModB | over GLRFIA | transporter activity |
| Mb2599c_2 | PCO_103 | 7.01 | -0.65 | 0.03545 | putative proteinc | over GLRFIA | Others |
| yheV | PCO_113 | 5.55 | -0.63 | 0.03696 | putative protein YheV | over GLRFIA | Others |
| modC | PCO_123 | 6.85 | -0.63 | 0.02836 | Molybdenum import ATP-binding protein ModC | over GLRFIA | Others |
| fadA_3 | PCO_127 | 6.15 | -0.60 | 0.03361 | Putative acyltransferase | over GLRFIA | others |
| PA1518 | PCO_98 | 5.36 | -0.58 | 0.03423 | 5-hydroxyisourate hydrolase | over GLRFIA | Others |
| R00369_1 | PCO_112 | 5.20 | -0.54 | 0.04274 | putative protein | over GLRFIA | others |
| prmC_2 | PCO_153 | 5.04 | 0.60 | 0.01804 | Release factor glutamine methyltransferase | over WT | Others |
| acoA | PCO_119 | 3.75 | 0.65 | 0.03057 | Acetoin:2%2C6-dichlorophenolindophenol oxidoreductase subunit alpha | over WT | Redox and Oxidative stress |
| fepC | PCO_103 | 6.10 | 0.66 | 0.03564 | Ferric enterobactin transport ATP-binding protein FepC | over WT | transporter activity |
| lysB | PCO_115 | 3.05 | 0.68 | 0.02595 | Protein lysB | over WT | Others |
| jayE | PCO_88 | 5.68 | 0.68 | 0.04552 | Putative protein JayE from lambdoid prophage e14 region | over WT | others |
| ydhJ_4 | PCO_85 | 3.85 | 0.69 | 0.02485 | putative protein YdhJ | over WT | others |
| hibch_2 | PCO_153 | 7.06 | 0.70 | 0.01021 | 3-hydroxyisobutyryl-CoA hydrolase%2C mitochondrial | over WT | aminoacid metabolism |
| mmgC_4 | PCO_153 | 6.93 | 0.70 | 0.00907 | Acyl-CoA dehydrogenase | over WT | fatty acid metabolism metabolism |
| 45 | PCO_88 | 4.29 | 0.70 | 0.03036 | Protein gp45 | over WT | Others |
| O | PCO_115 | 3.94 | 0.73 | 0.00224 | Presumed capsid-scaffolding protein | over WT | Others |
| 46 | PCO_88 | 3.95 | 0.75 | 0.03893 | Protein gp46 | over WT | others |
| ymfQ | PCO_88 | 4.88 | 0.78 | 0.00479 | putative protein YmfQ in lambdoid prophage e14 region | over WT | others |
| pfeA | PCO_103 | 10.86 | 0.80 | 0.04442 | Ferric enterobactin receptor precursor | over WT | membrane protein |
| nahR_4 | PCO_124 | 4.96 | 1.01 | 0.00081 | HTH-type transcriptional activator NahR | over WT | regulation of transcription. |
| gabP_2 | PCO_97 | 5.51 | 1.40 | 0.00000 | GABA permease | over WT | transporter activity |
